# Supplementary material for: Arabidopsis MYB47 and MYB95 transcription factors regulate jasmonate-inducible ER-body formation
Source: Commun Biol. 2025 Sep 26;8:1377. doi: 10.1038/s42003-025-08863-6 (PMC12475115; doi:10.1038/s42003-025-08863-6)
Supplement: Supplementary file 2 — Supplementary Information [file 42003_2025_8863_MOESM2_ESM.pdf]

Supplementary Information

Arabidopsis MYB47 and MYB95 Transcription Factors Regulate Jasmonate-inducible ER-Body Formation

Bizan J, Sarkar S, Basak AK, Bera S, Goto-Yamada S, Endo K, Tarnawska-Glatt K, Batth R, Bhardwaj K, Mirzaei M, Czerniawski P, Bednarek P, Yamada K.

Supplementary Figures 1 to 18
Supplementary Tables 1 to 9

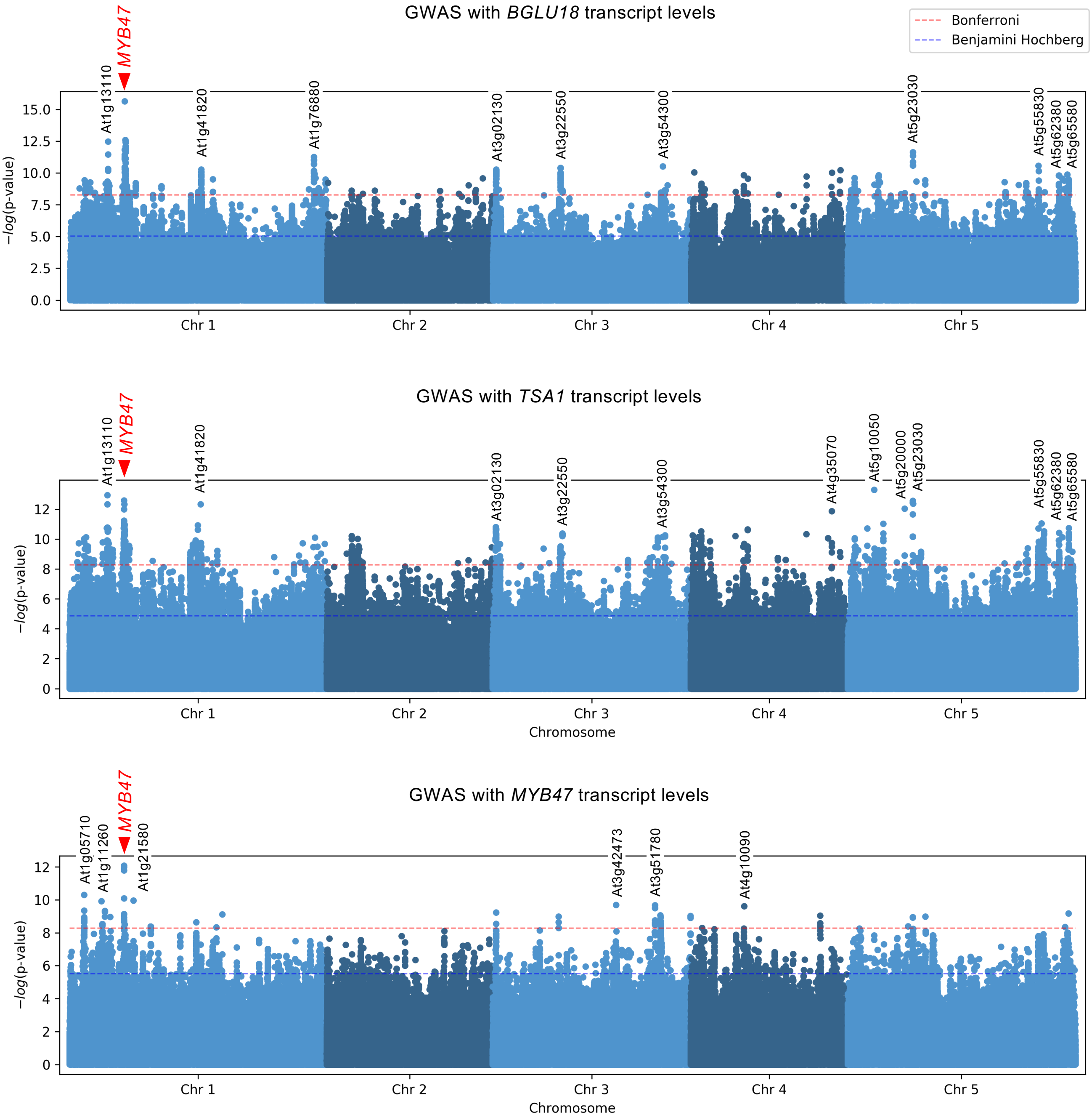

Supplementary Figure 1. Analysis of the correlation between *BGLU18*/*TSA1* expression and single nucleotide polymorphisms (SNPs) near the *MYB47* locus using genome-wide association study (GWAS). Manhattan plots were generated on the GWA-Portal (https://gwas.gmi.oeaw.ac.at) with the Kruskal-Wallis (KW) method using the SNP data from the 1001 Genomes dataset and no data transformation options. The expression of *BGLU18* and *TSA1* genes in Arabidopsis accessions was analyzed using publicly available data (see Methods for further details). Other genes with high *p*-value SNPs nearby are also indicated.

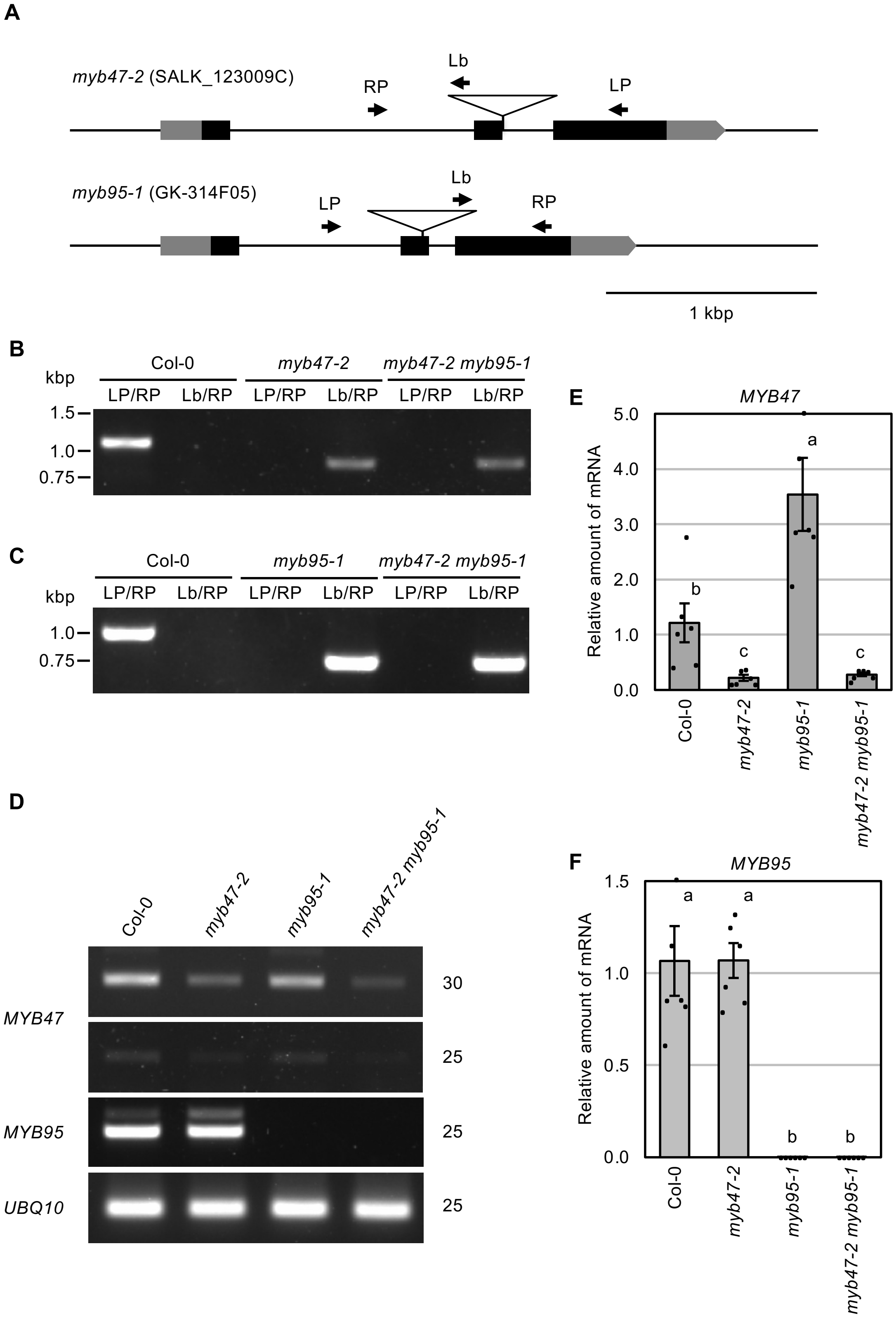

Supplementary Figure 2. Characterization of *myb47* and *myb95* mutants.

(A) Schematic diagram of the *myb47-2* and *myb95-1* gene structures. Exons and untranslated regions are represented by black and gray boxes, respectively. Triangles represent T-DNA insertion sites. Arrows indicate the positions and directions of PCR primers used for the PCR-based genotyping. Oligonucleotide sequences of the primers are listed in the Supplementary Table 8.

(B, C) PCR-based genotyping of the *myb47-2* **(B)** and *myb95-1* **(C)** mutants. Genome DNA was extracted from 10-day-old seedlings and subjected to PCR-based genotyping with the indicated primers.

(D) RT-PCR analysis of full-length *MYB47* and *MYB95* transcripts. *UBQ10* was used as an internal control to show the presence of cDNA in the PCR template. The numbers on the right indicate the number of PCR cycles.

(E, F) qRT-PCR analysis of *MYB47* **(E)** and *MYB95* **(F)**. Error bars indicate SE (n = 6 independent experiments). Different lowercase letters indicate significant differences ($p < 0.05$; Tukey's test).

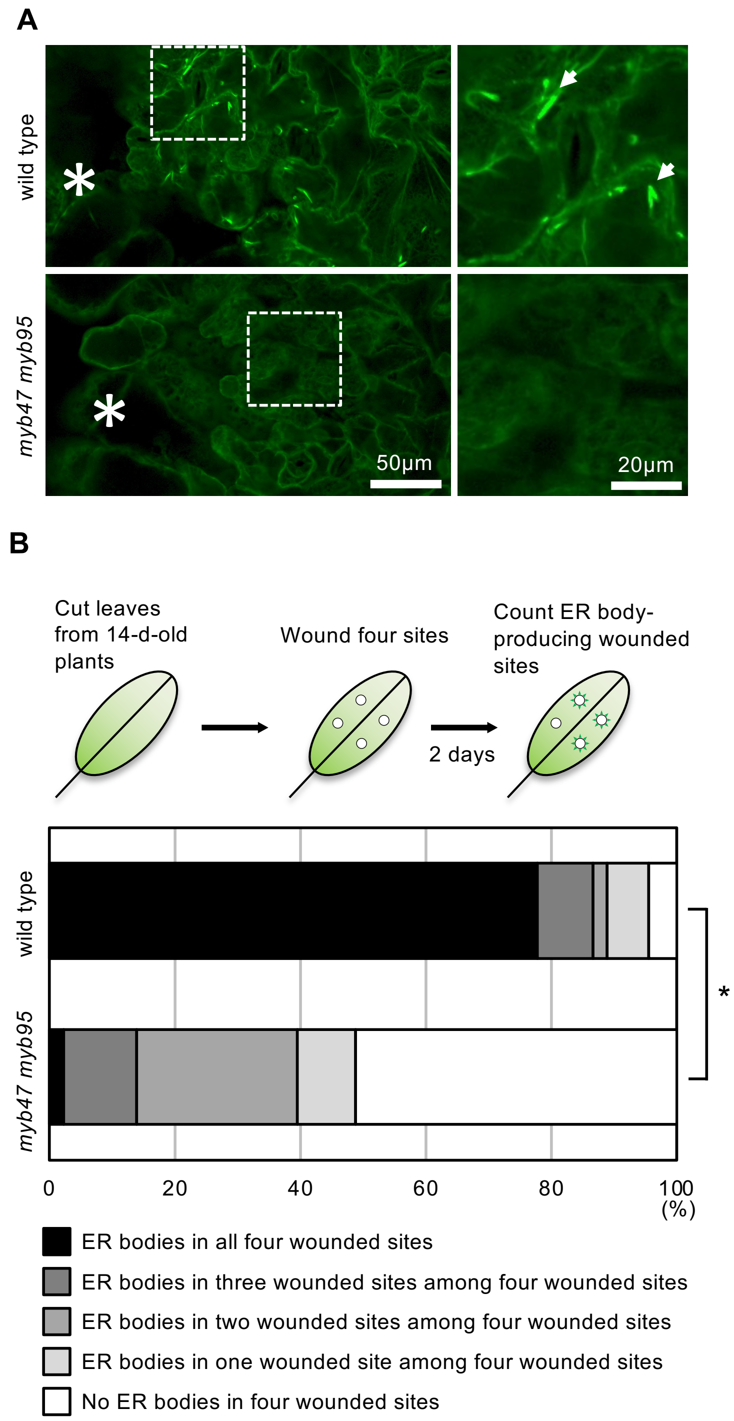

Supplementary Figure 3. MYB47 and MYB95 are required for the formation of wound-inducible ER bodies.

(A) Representative confocal laser scanning microscope images of leaves from stable transgenic plants in which ER and ER bodies are visualized with GFP. Rosette leaves from 14-day-old aseptically grown wild-type or *myb47,95* double mutant were wounded with a pipet tip, and the GFP signal was observed after 2 days. The GFP signal shows the ER and ER bodies. Asterisks indicate the wounded site, and arrows represent ER bodies.

(B) The chart shows the efficiency of wound-inducible ER body formation. The leaves were wounded with a pipet tip at four sites, and the number of wounded sites with forming ER bodies was counted per leaf under the epifluorescent microscope 2 days after wounding. The data are from n = 68 leaves for wild-type and n = 43 leaves for *myb47,95*. The asterisk denotes a significant difference in the data between wild-type and *myb47,95* ($p < 0.05$; Mann-Whitney U test).

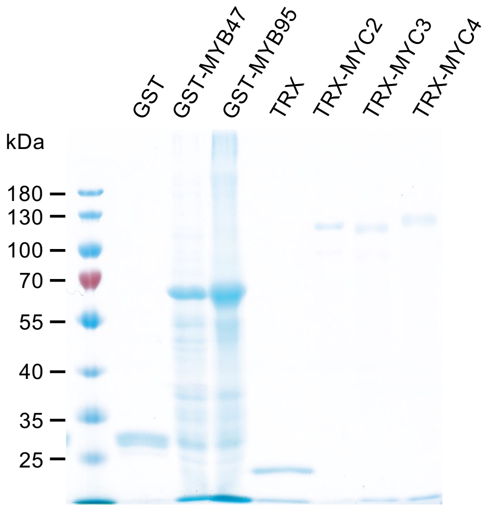

Supplementary Figure 4. Sodium dodecyl sulfate-polyacrylamide gel electrophoresis (SDS-PAGE) analysis of glutathione S-transferase (GST)- and thioredoxin (TRX)-tagged recombinant proteins purified from bacteria. The image shows an uncropped gel.

The affinity-purified GST- and TRX-tagged fusion proteins were subjected to SDS-PAGE, followed by Coomassie staining.

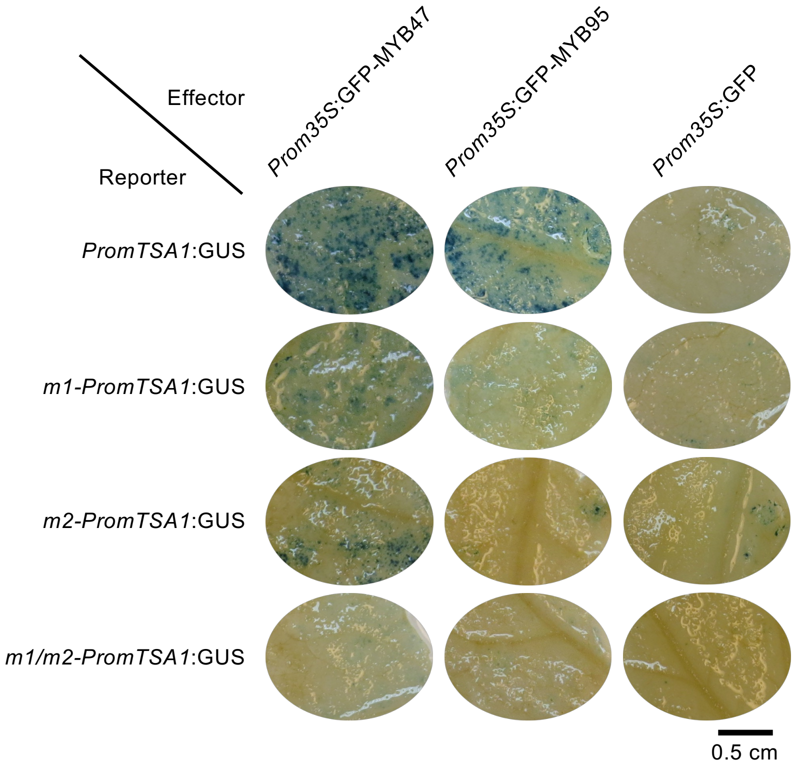

Supplementary Figure 5. Activation of the *TSA1* promoter by MYB47 and MYB95 in *Nicotiana benthamiana* leaves.

Agrobacterium harboring the effector construct was co-infiltrated into *N. benthamiana* leaves along with *Agrobacterium* harboring the reporter construct. Images show the GUS staining of *N. benthamiana* leaves. Scale bar = 0.5 cm.

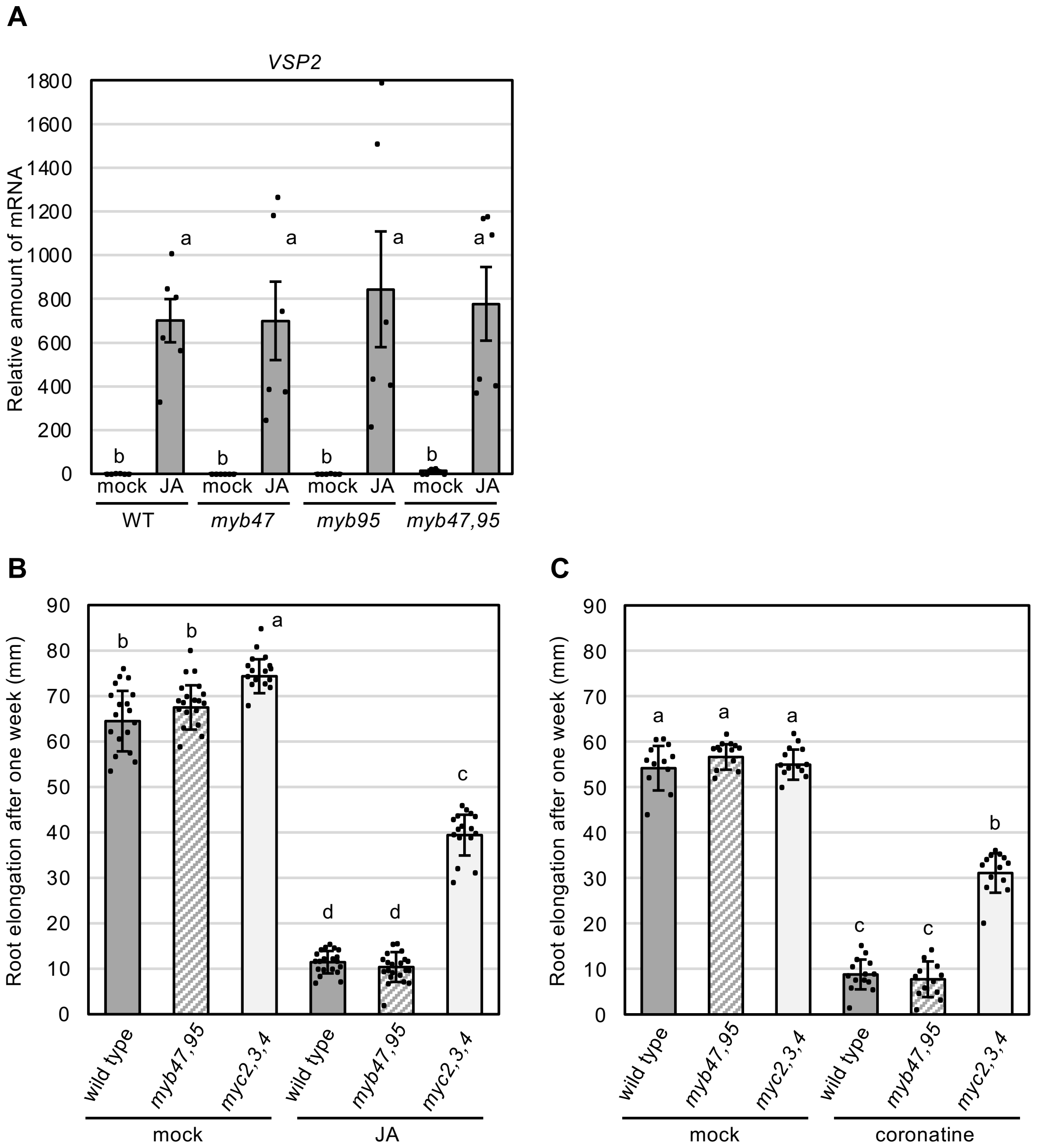

Supplementary Figure 6. Jasmonate (JA)-response phenotypes of the *myb47 myb95* double mutant.
(A) Expression analysis of *VSP2* in the rosette leaves of wild-type (WT), *myb47*, *myb95*, and *myb47,95* plants treated with or without JA for 2 days by qRT-PCR. Error bars represent SE (*n* = 6 independent experiments). Different lowercase letters indicate significant differences ($p < 0.05$; Tukey's test).
(B) Root elongation in wild-type, *myb47 myb95* double mutant, and *myc2,3,4* triple mutant plants grown on medium supplemented with (JA) or without (mock) 50 µM methyl jasmonate. Error bars represent SD (*n* = 16 to 21 plants). Different lowercase letters indicate significant differences ($p < 0.05$; Tukey's test).
(C) Root elongation in wild-type, *myb47,95* double mutant, and *myc2,3,4* triple mutant plants grown on medium supplemented with or without (mock) 50 µM coronatine. Error bars represent SD (*n* = 12 to 14 plants). Different lowercase letters indicate significant differences ($p < 0.05$; Tukey's test).

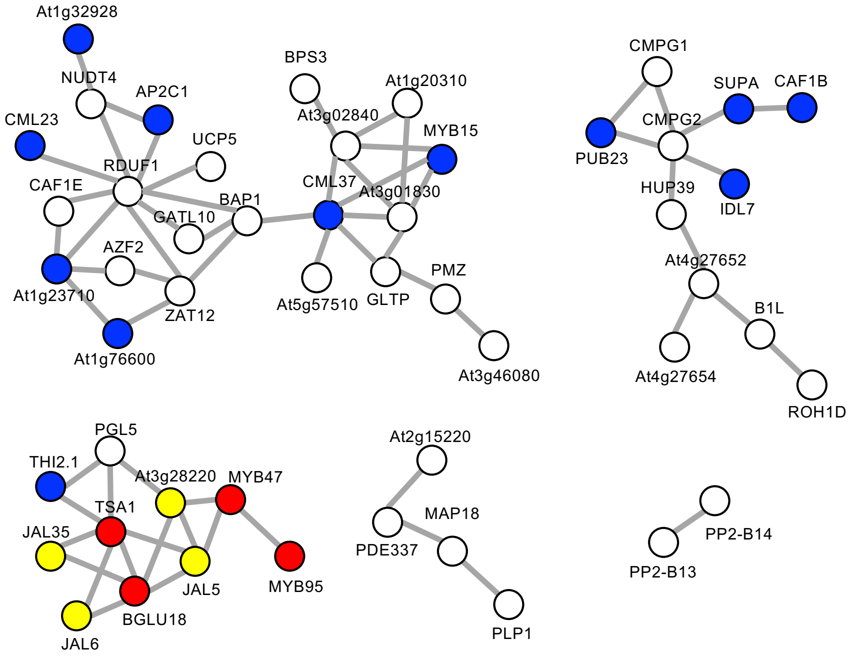

Supplementary Figure 7. Analysis of co-expressed gene clusters identified by searching ATTEDII database with *MYB47*, *MYB95*, and 77 MYB47/95-regulated genes.
The gene clusters show that their expressions are correlated to each other in the transcriptome database. Red circles represent *MYB47*, *MYB95*, *BGLU18*, and *TSA1*. Yellow circles represent genes whose products potentially form a protein complex with BGLU18. Blue circles indicate defense related genes. Nodes represent the correlation between co-expressed genes. Gene abbreviations are listed in Supplementary Table 5.

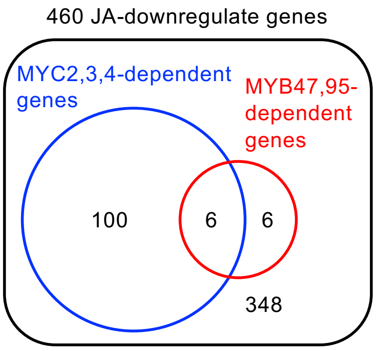

Supplementary Figure 8. Venn diagram of JA-repressed genes whose expressions were controlled by MYC2/3/4 and MYB47/95.

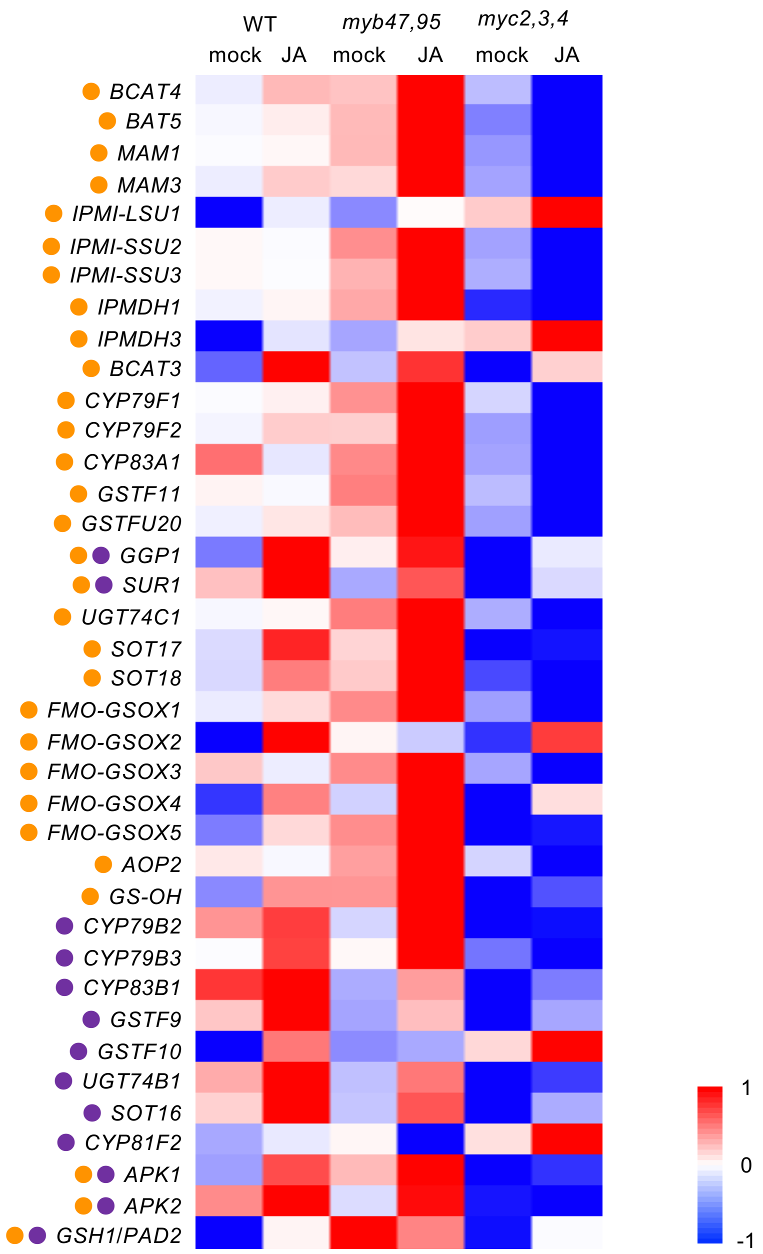

Supplementary Figure 9. Analysis of relative glucosinolate biosynthesis gene expression.
Red and blue colors indicate the highest and lowest gene expression levels within a row. Orange circles indicate aliphatic glucosinolate biosynthetic genes; purple circles indicate indolic glucosinolate biosynthetic genes.

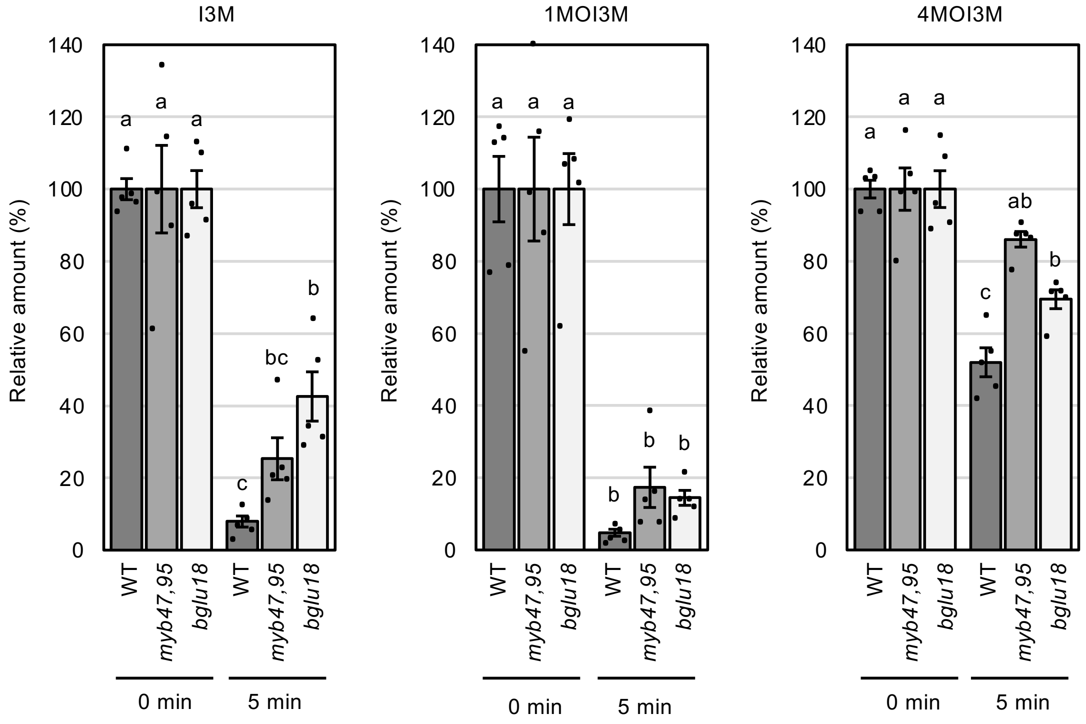

Supplementary Figure 10. Changes of indolic glucosinolate levels in the Arabidopsis leaf homogenate. Rosette leaves from 14-day-old aseptically-grown plants were cut and floated on 50 μM methyl jasmonate (JA) for 2 days and then homogenized. The glucosinolate levels in the homogenate were analysed immediately (0 min) or after incubation for 5 min at room temperature, which promotes degradation by the myrosinase activity. I3M, Indol-3-ylmethyl; 1MOI3M, 1-methoxyindol-3-ylmethyl; 4MOI3M, 4-methoxyindol-3-ylmethyl. Error bars indicate SE (n = 5 independent experiments). Different lowercase letters indicate significant differences ($p < 0.05$; Tukey's test).

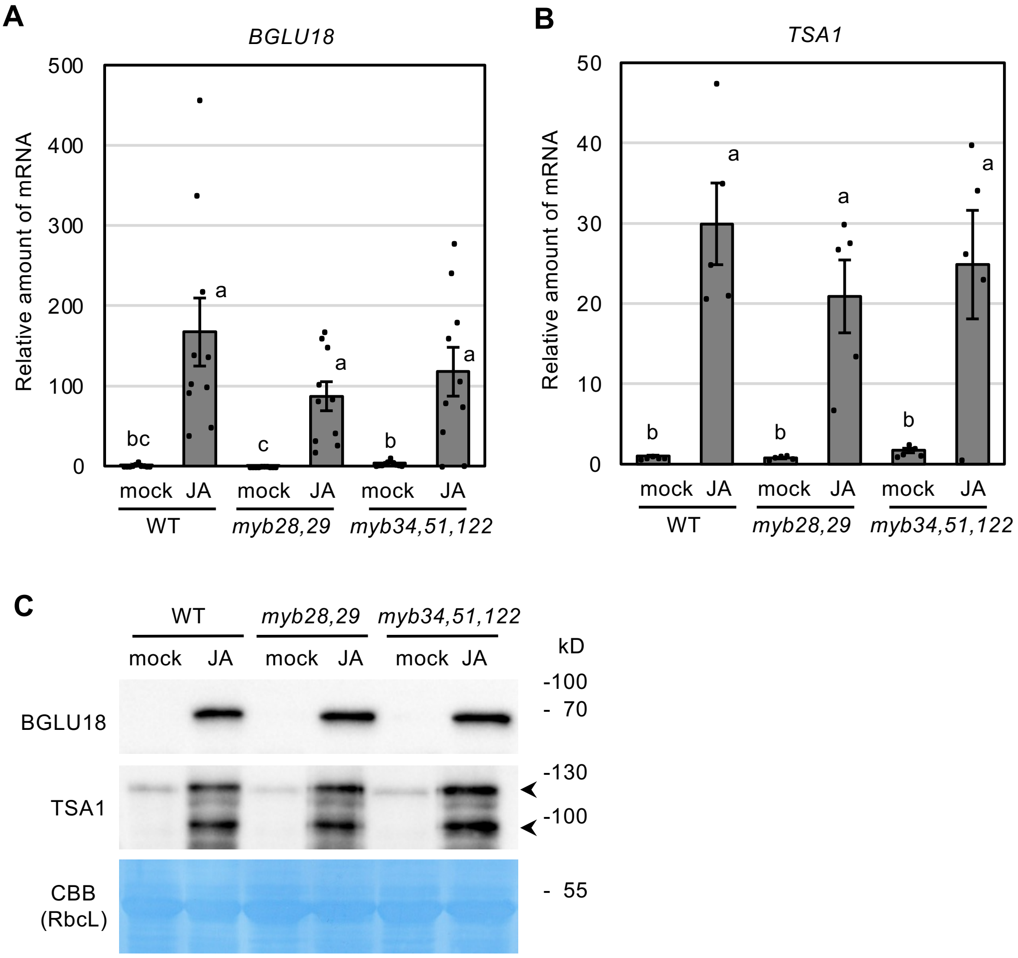

Supplementary Figure 11. JA treatment induces *BGLU18* and *TSA1* genes in *myb28,29* and *myb34,51,122* mutants.

(A) Induction of the *BGLU18* gene in Arabidopsis leaves. Rosette leaves were treated as described in (A). Rosette leaves from 14-day-old aseptically-grown wild-type (WT), *myb28,29* and *myb34,51,122* were cut and floated on distilled water (mock) or 50 μM methyl jasmonate (JA) for 2 days and then subjected to quantitative real-time PCR (qRT-PCR) analysis. Error bars represent SE (n = 10 independent experiments). Different lowercase letters indicate significant differences ($p < 0.05$; Tukey's test).

(B) Induction of the *TSA1* gene in Arabidopsis leaves. Error bars represent SE (n = 5 independent experiments). Different lowercase letters indicate significant differences ($p < 0.05$; Tukey's test).

(C) Accumulation of BGLU18 and TSA1 proteins in Arabidopsis leaves. Rosette leaves were treated as described in (A), and the extracted proteins were subjected to immunoblot analysis. Coomassie brilliant blue (CBB) staining shows the RuBisCo large subunit (RbcL) used as a loading control.

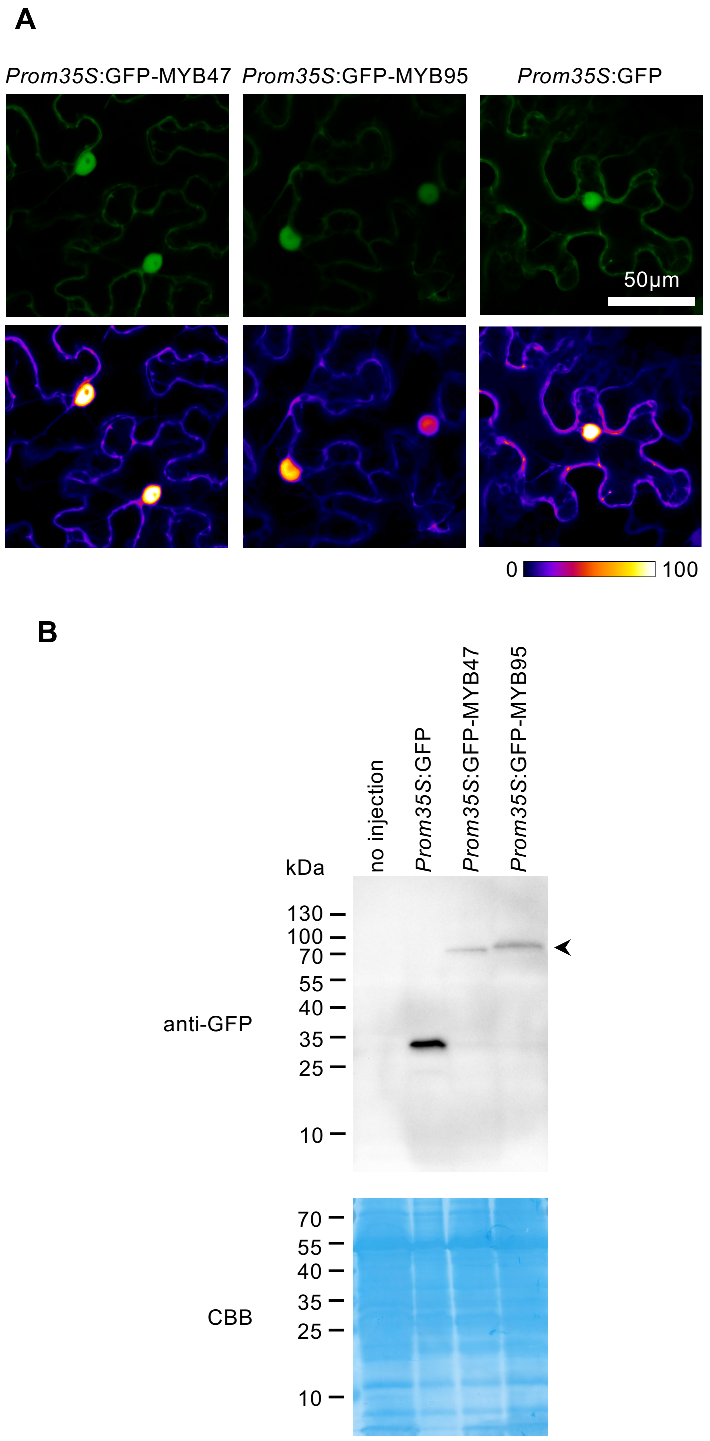

Supplementary Figure 12. Nuclear localization of MYB47 and MYB95.

(A) Confocal laser scanning microscope image showing the GFP signal in *N. benthamiana* leaves agroinfiltrated with the indicated constructs under the control of *35S* promoter. Lower panels show signal intensities calculated from the GFP signals of the upper panels. Scale bar = 50 µm.

(B) Immunoblot analysis of GFP-MYB47 and GFP-MYB95 fusion proteins extracted from *Nicotiana benthamiana* leaves. Proteins were extracted from leaves infiltrated with *Agrobacterium* harboring the indicated constructs and subjected to immunoblot analysis with anti-GFP antibody. The arrowhead indicates the GFP-MYB47 or GFP-MYB95 fusion protein. Coomassie staining shows the amount of protein loaded in each lane.

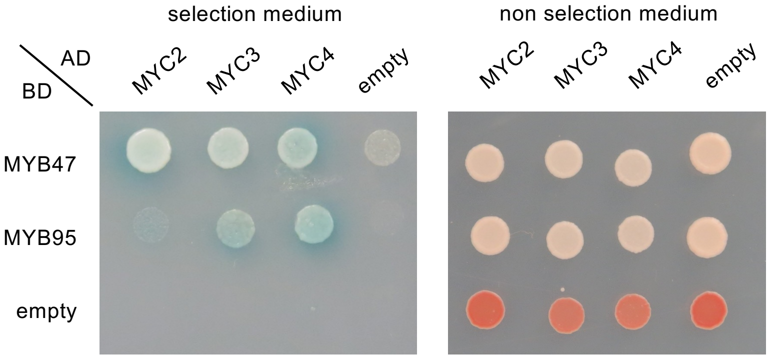

Supplementary Figure 13. Interaction between MYB and MYC proteins in the yeast two-hybrid assay. Constructs expressing the DNA-binding domain (BD)-MYB and activation domain (AD)-MYC fusion proteins were co-expressed in yeast, and protein–protein interactions were examined on the selection medium using a blue colorimetric β-galactosidase substrate. Empty vectors were used as a negative control.

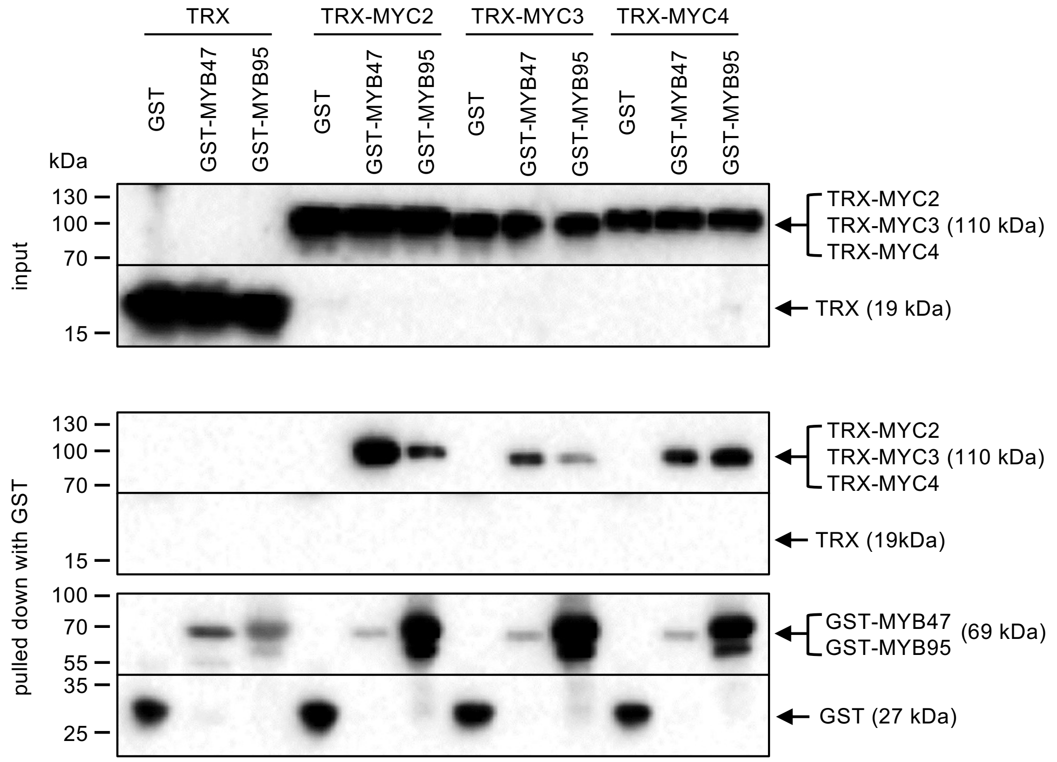

Supplementary Figure 14. Analysis of the protein-protein interaction between MYB and MYC proteins using *in vitro* pull-down assay.

Recombinant proteins expressed in *Escherichia coli* were used to perform the pull-down assay. The immunoblots show input and recovered protein complexes (pulled down with GST). The TRX- and GST-tagged proteins were detected with anti-6×His and anti-GST antibodies, respectively.

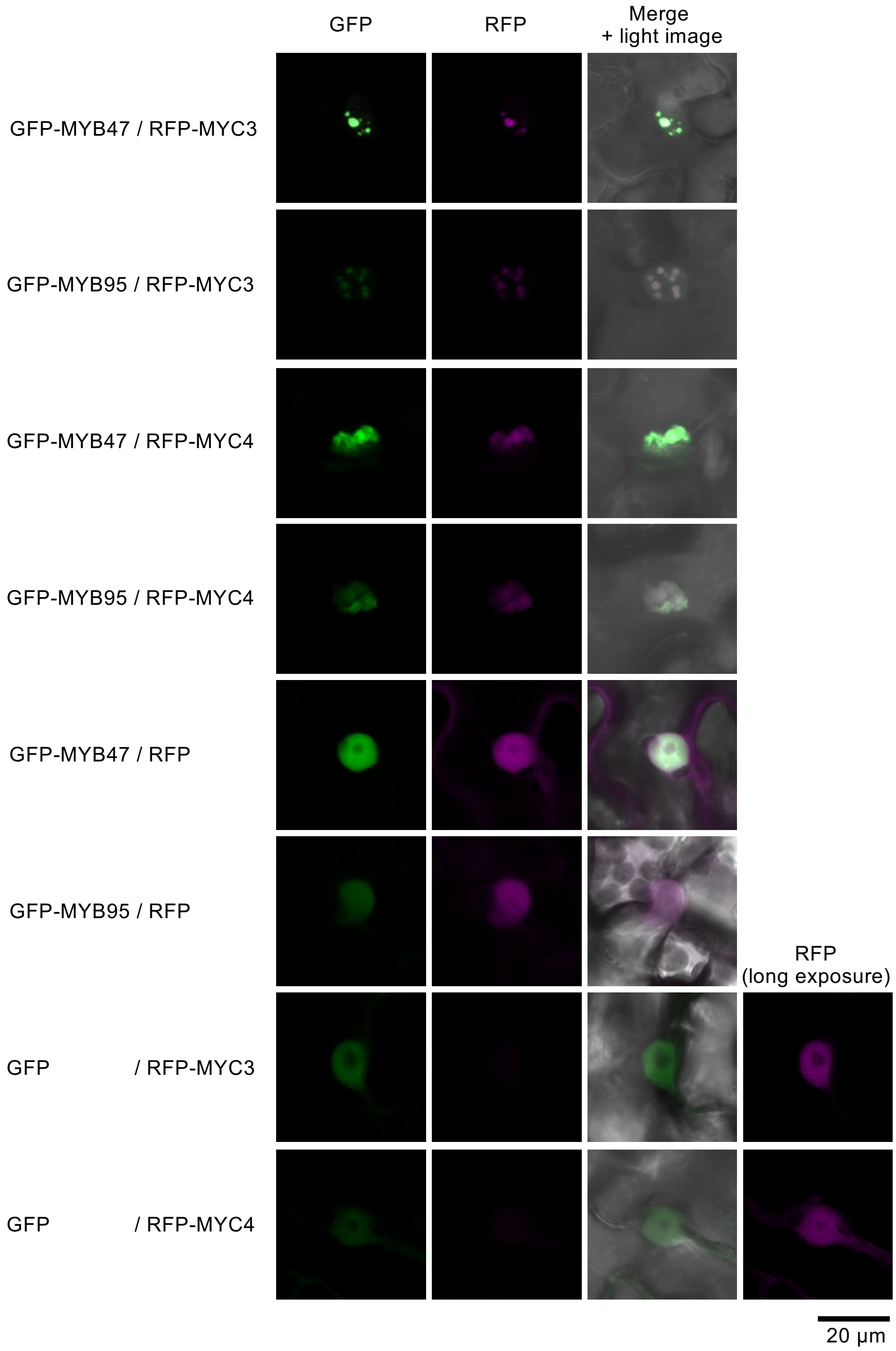

Supplementary Figure 15. Analysis of the co-localization of MYB47 and MYB95 proteins with MYC3 and MYC4 proteins.

Confocal laser scanning microscope images showing the co-localization of GFP-MYB and RFP-MYC fusion proteins in *N. benthamiana* leaves. All confocal images were taken with the same exposure time. Because of the low signal intensity of RFP in leaves co-expressing GFP with RFP-MYC3 or RFP-MYC4, images were also captured using longer exposure time.

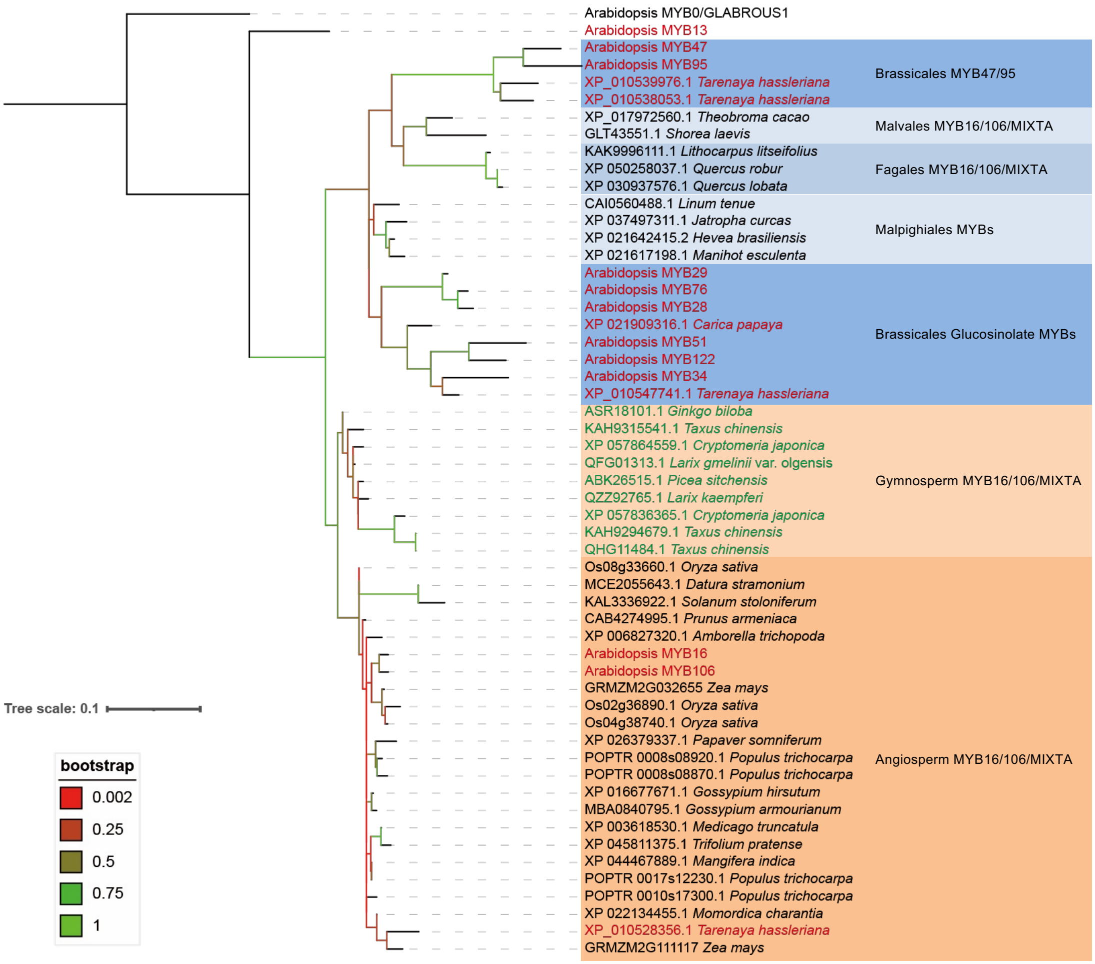

Supplementary Figure 16. Phylogenetic analysis of amino acid sequences of MYB16/106, MYB47/95, MYB28/29/76, and MYB34/51/122 homologs in gymnosperms and angiosperms.

Red characters show MYBs from Brassicales, and green characters show MYBs from gymnosperms. The bootstrap values are calculated with 500 iterations with the maximum-likelihood method and indicated as colors on the stem. Unrelated MYB0/GLABROUS1 was used to determine the root of the phylogenetic tree. Another member of subfamily VIII-D MYBs (Jiang and Rao, 2020), MYB13, was included to show MYB16/106, MYB47/95, MYB28/29/76, and MYB34/51/122 homologs constitute one clade in the subfamily VIII-D MYBs. Detailed information on proteins is listed in Supplementary Table 9.

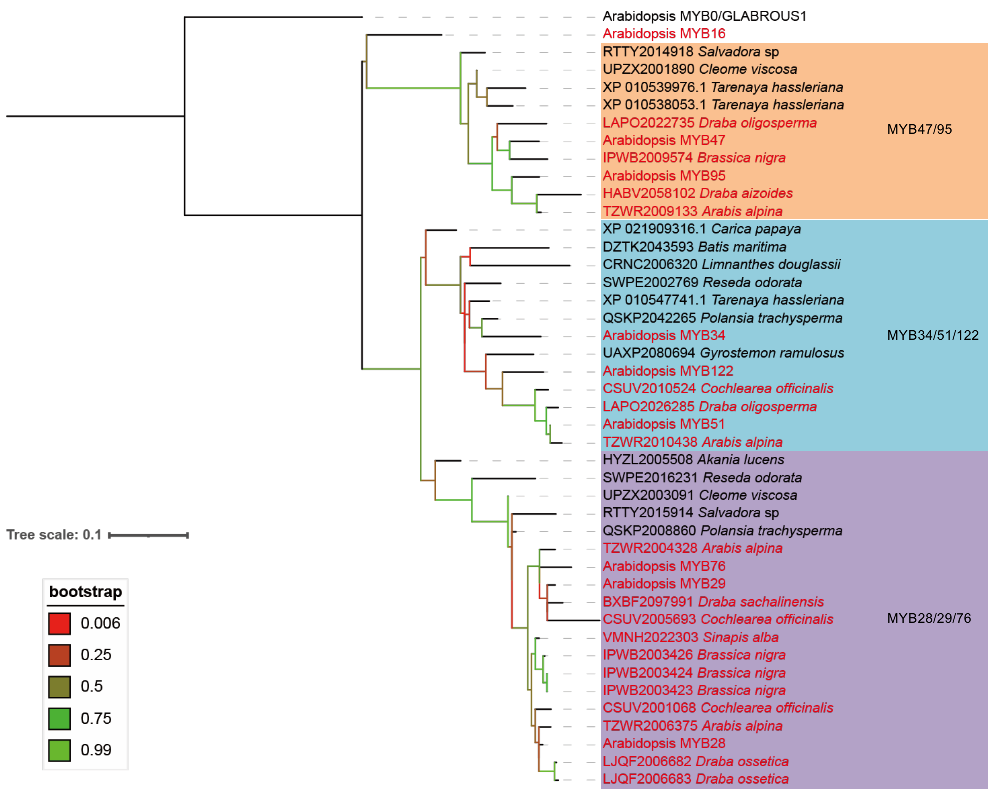

Supplementary Figure 17. Phylogenetic analysis of amino acid sequence of MYB47/95, MYB28/29/76, and MYB34/51/122 homologs in Brassicales.

Red characters show MYBs from Brassicaceae. The bootstrap values are calculated with 500 iterations with the maximum-likelihood method and indicated as colors on the stem. The 1000 Plant transcriptome database was searched to gain the protein data. Unrelated MYB0/GLABROUS1 was used to determine the root of the phylogenetic tree. Detailed information on proteins is listed in Supplementary Table 9.

Figure 1c

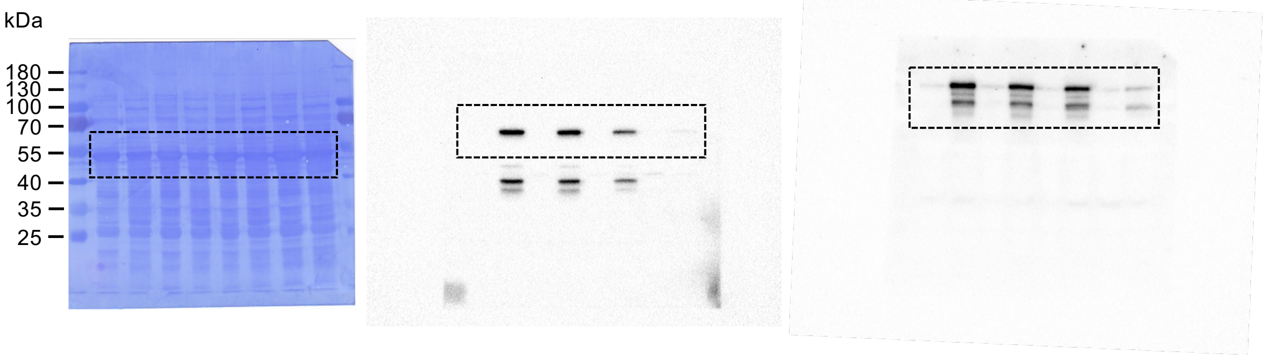

Figure 3b

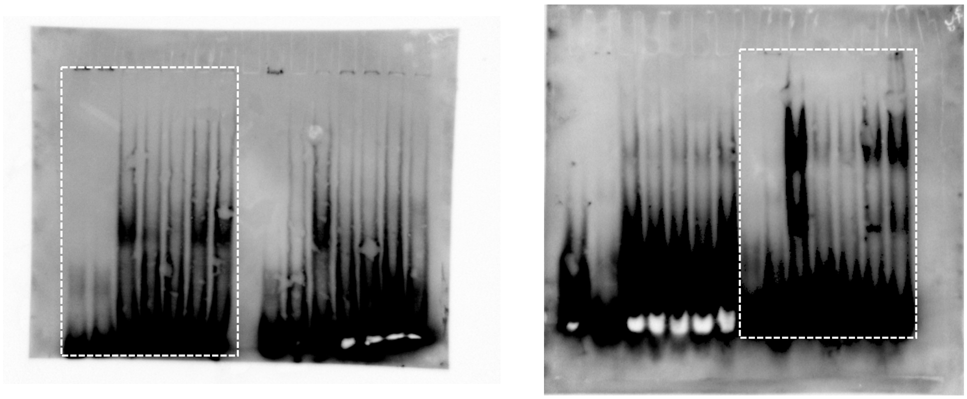

Figure 6b

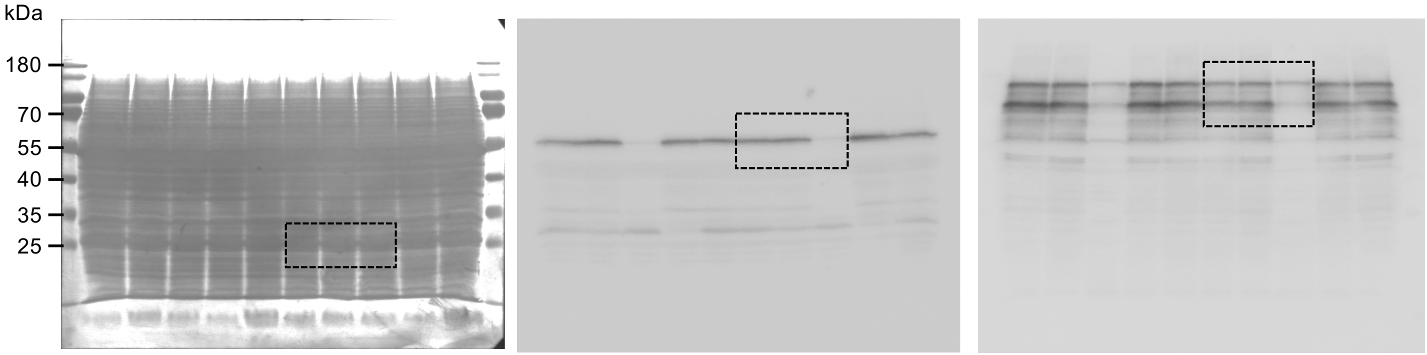

Figure 8b

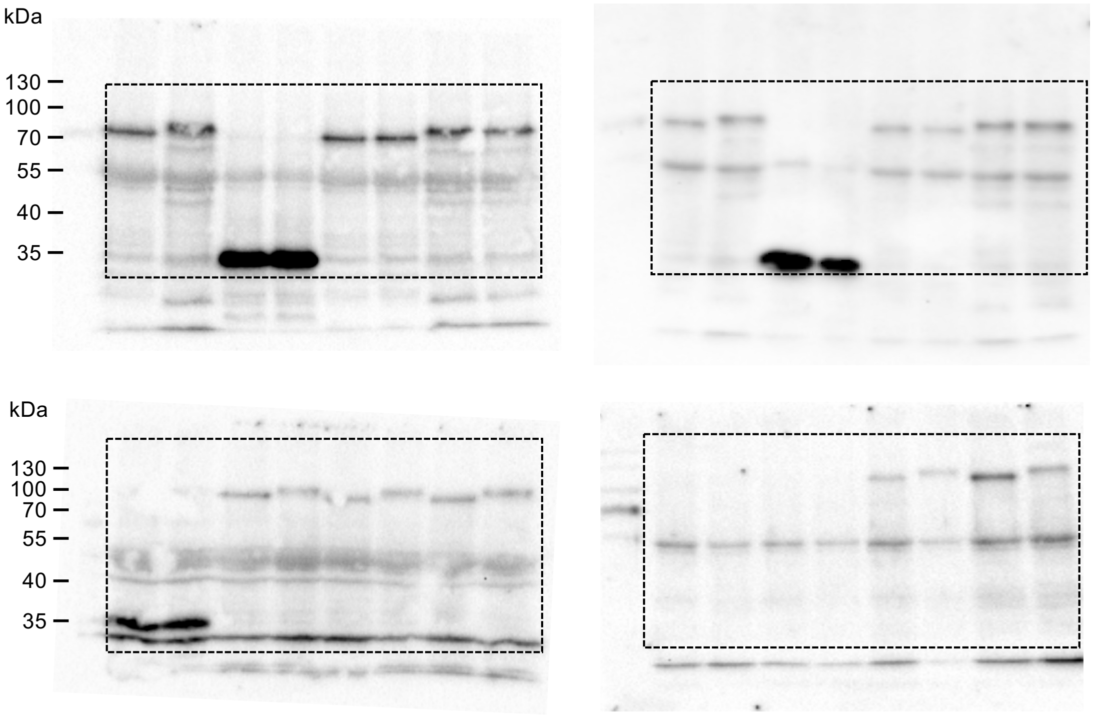

Supplementary Figure 18. Uncropped blot and gel images.

Supplementary Figure 2

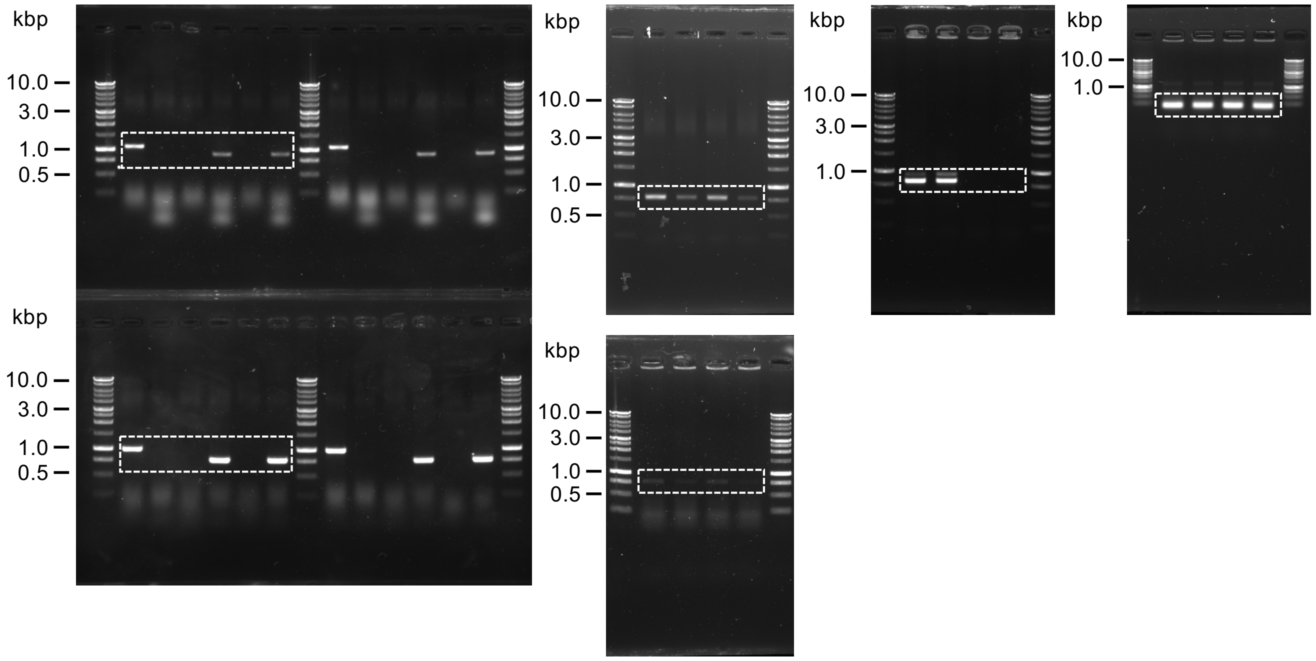

Supplementary Figure 11

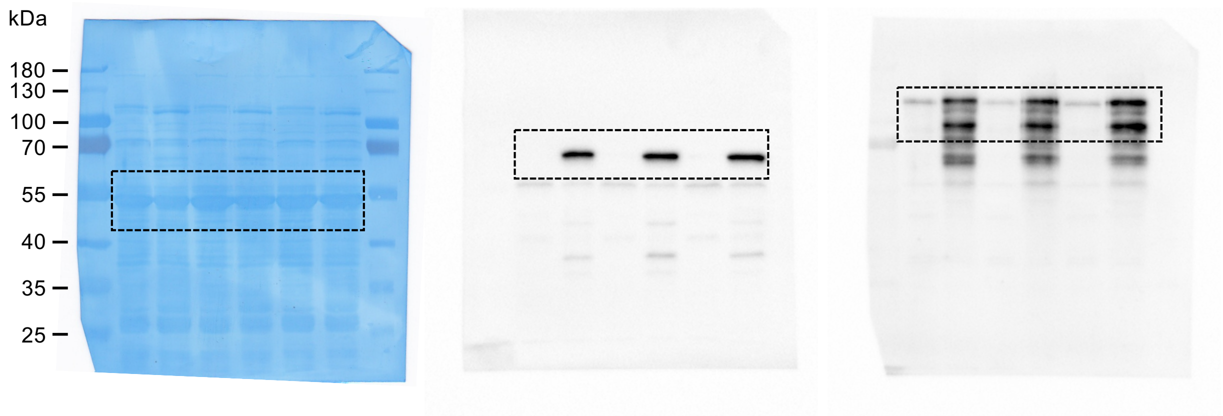

Supplementary Figure 12

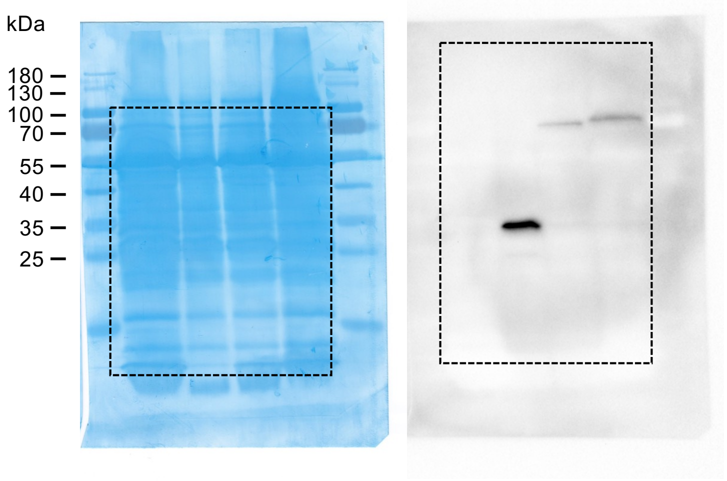

Supplementary Figure 18. Uncropped blot and gel images.

Supplementary Figure 14

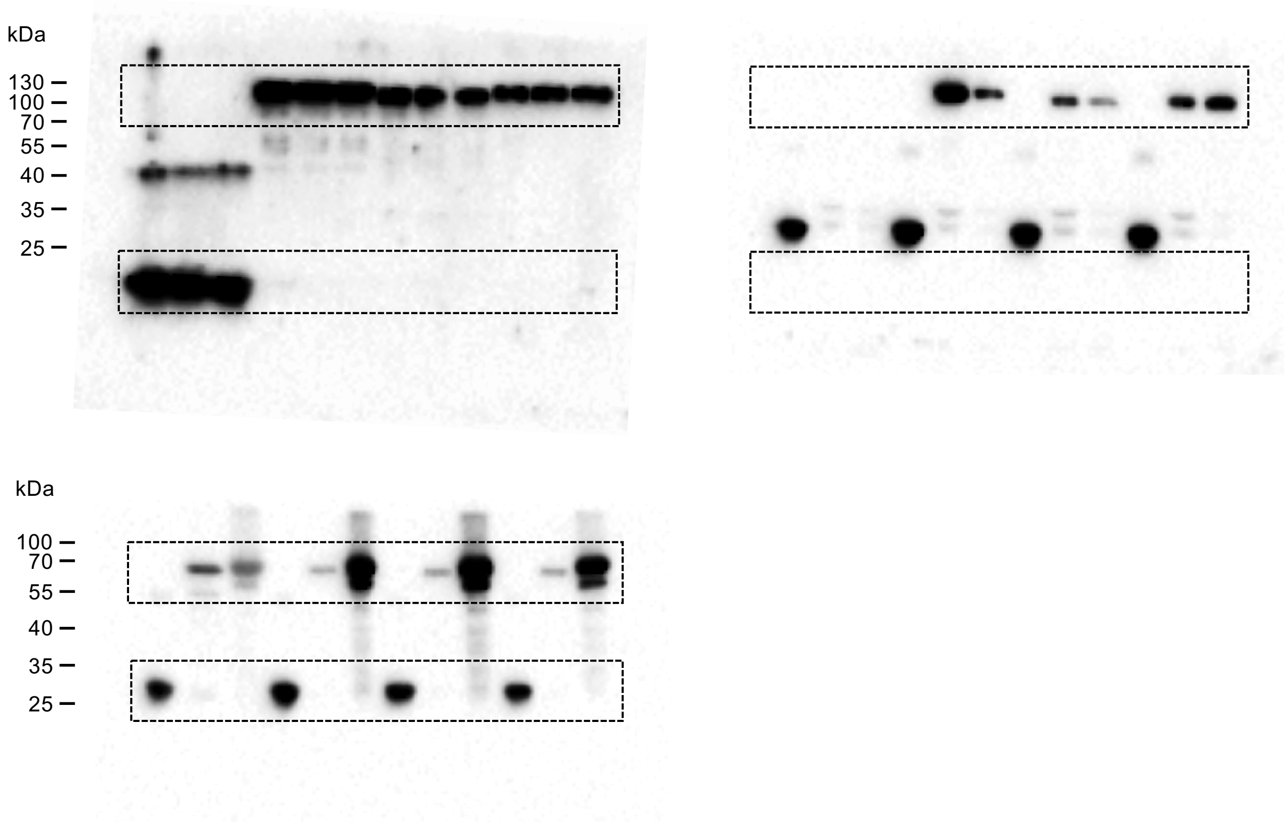

Supplementary Figure 18. Uncropped blot and gel images.

Supplementary Table 1. List of the genes that are co-expressed with *BGLU18* according to ATTED-II database.

Rank*	Score	Locus	Gene	Function
0	14.2	AT1G52400	BGLU18	beta glucosidase 18
1	14.2	AT1G52410	TSA1	TSK-associating protein 1
2	12.5	AT1G52000	AT1G52000	Mannose-binding lectin superfamily protein
3	11.5	AT3G28220	AT3G28220	TRAF-like family protein
4	11.4	AT1G52040	MBP1	myrosinase-binding protein 1
5	10.8	AT1G52030	MBP2	myrosinase-binding protein 2
6	10.8	AT2G39330	JAL23	jacalin-related lectin 23
7	10.4	AT1G24070	CSLA10	cellulose synthase-like A10
8	10.4	AT3G45140	LOX2	lipoxygenase 2
9	10.0	AT1G54020	AT1G54020	GDSL-like Lipase/Acylhydrolase superfamily protein
10	9.0	AT2G43530	AT2G43530	scorpion toxin-like knottin superfamily protein
11	8.9	AT3G16470	JR1	Mannose-binding lectin superfamily protein
12	8.8	AT5G24420	PGL5	6-phosphogluconolactonase 5
13	8.7	AT1G72260	THI2.1	thionin 2.1
14	8.5	AT1G19670	CLH1	chlorophyllase 1
15	8.2	AT1G78490	CYP708A3	cytochrome P450: family 708: subfamily A: polypeptide 3
16	7.8	AT4G23600	CORI3	Tyrosine transaminase family protein
17	7.3	AT1G31550	AT1G31550	GDSL-like Lipase/Acylhydrolase superfamily protein
18	7.3	AT2G34810	AT2G34810	FAD-binding Berberine family protein
19	7.2	AT5G02940	AT5G02940	ion channel POLLUX-like protein: putative (DUF1012)
20	7.1	AT4G17470	AT4G17470	alpha/beta-Hydrolases superfamily protein
21	6.6	AT5G42650	AOS	allene oxide synthase
22	6.4	AT4G15210	BAM5	beta-amylase 5
23	6.4	AT4G15440	HPL1	hydroperoxide lyase 1
24	6.2	AT3G12145	FLR1	Leucine-rich repeat (LRR) family protein
25	6.1	AT5G24770	VSP2	vegetative storage protein 2
26	5.9	AT5G06870	PGIP2	polygalacturonase inhibiting protein 2
27	5.9	AT5G24780	VSP1	vegetative storage protein 1
28	5.9	AT4G13410	ATCSLA15	Nucleotide-diphospho-sugar transferases superfamily protein
29	5.6	AT3G25770	AOC2	allene oxide cyclase 2
30	5.5	AT1G74430	MYB95	myb domain protein 95
31	5.5	AT3G25760	AOC1	allene oxide cyclase 1
32	5.4	AT1G54040	ESP	epithiospecifier protein
33	5.4	AT4G21910	AT4G21910	MATE efflux family protein
34	5.4	AT3G51450	AT3G51450	Calcium-dependent phosphotriesterase superfamily protein
35	5.4	AT1G18710	MYB47	myb domain protein 47
36	5.4	AT4G24350	AT4G24350	Phosphorylase superfamily protein
37	5.2	AT2G43550	AT2G43550	scorpion toxin-like knottin superfamily protein
38	5.1	AT1G24020	MLP423	MLP-like protein 423
39	5.1	AT1G19570	DHAR1	dehydroascorbate reductase
40	5.0	AT2G46640	AT2G46640	NAD-dependent protein deacetylase HST1-like protein
41	4.9	AT2G39030	NATA1	Acyl-CoA N-acyltransferases (NAT) superfamily protein
42	4.9	AT4G18440	AT4G18440	L-Aspartase-like family protein
43	4.9	AT1G62540	FMO GS-OX2	flavin-monooxygenase glucosinolate S-oxygenase 2
44	4.7	AT1G68600	AT1G68600	aluminum activated malate transporter family protein
45	4.7	AT1G14250	AT1G14250	GDA1/CD39 nucleoside phosphatase family protein
46	4.7	AT1G70830	MLP28	MLP-like protein 28
47	4.6	AT2G23010	SCPL9	serine carboxypeptidase-like 9
48	4.5	AT5G41120	AT5G41120	Esterase/lipase/thioesterase family protein
49	4.5	AT5G52320	CYP96A4	cytochrome P450: family 96: subfamily A: polypeptide 4
50	4.5	AT1G73325	AT1G73325	Kunitz family trypsin and protease inhibitor protein

*, top 50 coexpressed genes.

Supplementary Table 2. List of the genes that are co-expressed with *TSA1* according to ATTED-II database.

Rank*	Score	Locus	Gene	Function
0	14.2	AT1G52410	TSA1	TSK-associating protein 1
1	14.2	AT1G52400	BGLU18	beta glucosidase 18
2	12.2	AT1G52000	AT1G52000	Mannose-binding lectin superfamily protein
3	12.0	AT2G39330	JAL23	jacalin-related lectin 23
4	11.1	AT3G16470	JR1	Mannose-binding lectin superfamily protein
5	10.5	AT1G52040	MBP1	myrosinase-binding protein 1
6	10.4	AT1G54020	AT1G54020	GDSL-like Lipase/Acylhydrolase superfamily protein
7	10.1	AT1G52030	MBP2	myrosinase-binding protein 2
8	9.8	AT3G28220	AT3G28220	TRAF-like family protein
9	9.6	AT1G24070	CSLA10	cellulose synthase-like A10
10	8.9	AT3G45140	LOX2	lipoxygenase 2
11	8.8	AT2G43530	AT2G43530	scorpion toxin-like knottin superfamily protein
12	8.5	AT1G72260	THI2.1	thionin 2.1
13	8.4	AT2G34810	AT2G34810	FAD-binding Berberine family protein
14	8.3	AT1G78490	CYP708A3	cytochrome P450: family 708: subfamily A: polypeptide 3
15	8.3	AT5G24420	PGL5	6-phosphogluconolactonase 5
16	8.2	AT1G19670	CLH1	chlorophyllase 1
17	7.6	AT1G31550	AT1G31550	GDSL-like Lipase/Acylhydrolase superfamily protein
18	7.3	AT4G17470	AT4G17470	alpha/beta-Hydrolases superfamily protein
19	6.9	AT4G23600	CORI3	Tyrosine transaminase family protein
20	6.8	AT5G02940	AT5G02940	ion channel POLLUX-like protein: putative (DUF1012)
21	6.7	AT4G15210	BAM5	beta-amylase 5
22	6.6	AT5G24770	VSP2	vegetative storage protein 2
23	6.5	AT5G42650	AOS	allene oxide synthase
24	6.5	AT5G24780	VSP1	vegetative storage protein 1
25	6.4	AT4G21910	AT4G21910	MATE efflux family protein
26	6.3	AT3G51450	AT3G51450	Calcium-dependent phosphotriesterase superfamily protein
27	6.1	AT4G15440	HPL1	hydroperoxide lyase 1
28	6.1	AT3G48350	CEP3	Cysteine proteinases superfamily protein
29	6.0	AT5G41120	AT5G41120	Esterase/lipase/thioesterase family protein
30	6.0	AT1G74430	MYB95	myb domain protein 95
31	5.9	AT4G29700	AT4G29700	Alkaline-phosphatase-like family protein
32	5.9	AT1G19570	DHAR1	dehydroascorbate reductase
33	5.8	AT3G15790	MBD11	methyl-CPG-binding domain 11
34	5.8	AT4G13410	ATCSLA15	Nucleotide-diphospho-sugar transferases superfamily protein
35	5.7	AT5G06870	PGIP2	polygalacturonase inhibiting protein 2
36	5.7	AT2G34490	CYP710A2	cytochrome P450: family 710: subfamily A: polypeptide 2
37	5.7	AT3G12145	FLR1	Leucine-rich repeat (LRR) family protein
38	5.6	AT2G20340	AAS	Pyridoxal phosphate (PLP)-dependent transferases superfamily protein
39	5.6	AT5G52320	CYP96A4	cytochrome P450: family 96: subfamily A: polypeptide 4
40	5.5	AT4G18440	AT4G18440	L-Aspartase-like family protein
41	5.5	AT2G38750	ANNAT4	annexin 4
42	5.4	AT2G43550	AT2G43550	scorpion toxin-like knottin superfamily protein
43	5.4	AT2G39030	NATA1	Acyl-CoA N-acyltransferases (NAT) superfamily protein
44	5.4	AT3G25760	AOC1	allene oxide cyclase 1
45	5.3	AT5G65280	GCL1	GCR2-like 1
46	5.2	AT1G18710	MYB47	myb domain protein 47
47	5.2	AT5G23820	AT5G23820	MD-2-related lipid recognition domain-containing protein
48	5.1	AT4G24350	AT4G24350	Phosphorylase superfamily protein
49	5.1	AT1G62540	FMO GS-OX2	flavin-monooxygenase glucosinolate S-oxygenase 2
50	5.0	AT1G65890	AAE12	acyl activating enzyme 12

*, top 50 coexpressed genes.

Supplementary Table 3. List of the genes that are co-expressed with *MYB47* according to ATTED-II database.

Rank*	Score	Locus	Gene	Function
0	14.2	AT1G18710	MYB47	myb domain protein 47
1	10.9	AT1G52000	AT1G52000	Mannose-binding lectin superfamily protein
2	7.1	AT1G74430	MYB95	myb domain protein 95
3	6.9	AT3G28220	AT3G28220	TRAF-like family protein
4	6.7	AT4G15440	HPL1	hydroperoxide lyase 1
5	5.8	AT3G28270	AT3G28270	transmembrane protein: putative (DUF677)
6	5.7	AT5G02940	AT5G02940	ion channel POLLUX-like protein: putative (DUF1012)
7	5.7	AT1G19670	CLH1	chlorophyllase 1
8	5.6	AT5G42650	AOS	allene oxide synthase
9	5.6	AT1G52040	MBP1	myrosinase-binding protein 1
10	5.4	AT1G52400	BGLU18	beta glucosidase 18
11	5.4	AT5G52320	CYP96A4	cytochrome P450: family 96: subfamily A: polypeptide 4
12	5.2	AT1G52410	TSA1	TSK-associating protein 1
13	5.2	AT1G56650	PAP1	production of anthocyanin pigment 1
14	5.2	AT5G67180	TOE3	target of early activation tagged (EAT) 3
15	5.0	AT1G54160	NF-YA5	nuclear factor Y: subunit A5
16	5.0	AT2G40435	AT2G40435	transcription factor SCREAM-like protein
17	4.9	AT3G27250	AT3G27250	hypothetical protein
18	4.8	AT2G43530	AT2G43530	scorpion toxin-like knottin superfamily protein
19	4.7	AT4G23600	CORI3	Tyrosine transaminase family protein
20	4.7	AT2G42540	COR15A	cold-regulated 15a
21	4.6	AT1G24070	CSLA10	cellulose synthase-like A10
22	4.6	AT3G25760	AOC1	allene oxide cyclase 1
23	4.6	AT1G65890	AAE12	acyl activating enzyme 12
24	4.5	AT2G20340	AAS	Pyridoxal phosphate (PLP)-dependent transferases superfamily protein
25	4.5	AT5G61810	APC1	Mitochondrial substrate carrier family protein
26	4.5	AT1G31550	AT1G31550	GDSL-like Lipase/Acylhydrolase superfamily protein
27	4.4	AT3G45140	LOX2	lipoxygenase 2
28	4.4	AT1G01250	AT1G01250	Integrase-type DNA-binding superfamily protein
29	4.3	AT1G56600	GolS2	galactinol synthase 2
30	4.3	AT2G46640	AT2G46640	NAD-dependent protein deacetylase HST1-like protein
31	4.3	AT1G19650	AT1G19650	Sec14p-like phosphatidylinositol transfer family protein
32	4.3	AT1G28230	PUP1	purine permease 1
33	4.3	AT2G39250	SNZ	Integrase-type DNA-binding superfamily protein
34	4.2	AT2G46510	AIB	ABA-inducible BHLH-type transcription factor
35	4.1	AT3G19620	AT3G19620	Glycosyl hydrolase family protein
36	4.1	AT1G24330	AT1G24330	ARM repeat superfamily protein
37	4.1	AT1G54040	ESP	epithiospecifier protein
38	4.1	AT1G68600	AT1G68600	aluminum activated malate transporter family protein
39	4.1	AT1G76955	AT1G76955	Expressed protein
40	4.0	AT4G37980	ELI3-1	cinnamyl alcohol dehydrogenase 7
41	4.0	AT2G38750	ANNAT4	annexin 4
42	4.0	AT3G15790	MBD11	methyl-CPG-binding domain 11
43	4.0	AT1G72450	JAZ6	jasmonate-zim-domain protein 6
44	4.0	AT5G02950	AT5G02950	Tudor/PWWP/MBT superfamily protein
45	3.9	AT2G39800	P5CS1	delta1-pyrroline-5-carboxylate synthase 1
46	3.9	AT5G47640	NF-YB2	nuclear factor Y: subunit B2
47	3.9	AT1G80130	AT1G80130	Tetratricopeptide repeat (TPR)-like superfamily protein
48	3.9	AT5G24420	PGL5	6-phosphogluconolactonase 5
49	3.8	AT5G03190	CPUORF47	peptide upstream protein
50	3.8	AT1G70700	TIFY7	TIFY domain/Divergent CCT motif family protein

*, top 50 coexpressed genes.

Supplementary Table 4. List of the genes that are co-expressed with *MYB95* according to ATTED-II database.

Rank*	Score	Locus	Gene	Function
0	14.2	AT1G74430	MYB95	myb domain protein 95
1	7.3	AT1G52000	AT1G52000	Mannose-binding lectin superfamily protein
2	7.2	AT1G31550	AT1G31550	GDSL-like Lipase/Acylhydrolase superfamily protein
3	7.1	AT5G52320	CYP96A4	cytochrome P450: family 96: subfamily A: polypeptide 4
4	7.1	AT1G18710	MYB47	myb domain protein 47
5	7.1	AT1G19670	CLH1	chlorophyllase 1
6	6.9	AT2G46510	AIB	ABA-inducible BHLH-type transcription factor
7	6.9	AT1G17420	LOX3	lipoxygenase 3
8	6.7	AT1G17380	JAZ5	jasmonate-zim-domain protein 5
9	6.5	AT2G43530	AT2G43530	scorpion toxin-like knottin superfamily protein
10	6.4	AT2G06050	OPR3	oxophytodienoate-reductase 3
11	6.3	AT5G14700	AT5G14700	NAD(P)-binding Rossmann-fold superfamily protein
12	6.0	AT1G52410	TSA1	TSK-associating protein 1
13	5.9	AT3G51450	AT3G51450	Calcium-dependent phosphotriesterase superfamily protein
14	5.8	AT5G02940	AT5G02940	ion channel POLLUX-like protein: putative (DUF1012)
15	5.8	AT4G15440	HPL1	hydroperoxide lyase 1
16	5.8	AT3G47960	GTR1	Major facilitator superfamily protein
17	5.8	AT2G42760	AT2G42760	DUF1685 family protein
18	5.8	AT1G20510	OPCL1	OPC-8:0 CoA ligase1
19	5.8	AT1G24330	AT1G24330	ARM repeat superfamily protein
20	5.8	AT1G72450	JAZ6	jasmonate-zim-domain protein 6
21	5.7	AT1G70700	TIFY7	TIFY domain/Divergent CCT motif family protein
22	5.6	AT1G65890	AAE12	acyl activating enzyme 12
23	5.5	AT2G39330	JAL23	jacalin-related lectin 23
24	5.5	AT5G24420	PGL5	6-phosphogluconolactonase 5
25	5.5	AT5G42650	AOS	allene oxide synthase
26	5.5	AT1G52400	BGLU18	beta glucosidase 18
27	5.2	AT5G13220	JAZ10	jasmonate-zim-domain protein 10
28	5.2	AT1G74950	TIFY10B	TIFY domain/Divergent CCT motif family protein
29	5.1	AT1G24070	CSLA10	cellulose synthase-like A10
30	5.1	AT5G06870	PGIP2	polygalacturonase inhibiting protein 2
31	5.1	AT1G72520	LOX4	PLAT/LH2 domain-containing lipoxygenase family protein
32	5.0	AT1G30135	JAZ8	jasmonate-zim-domain protein 8
33	5.0	AT1G78490	CYP708A3	cytochrome P450: family 708: subfamily A: polypeptide 3
34	4.9	AT2G34600	JAZ7	jasmonate-zim-domain protein 7
35	4.9	AT1G61890	AT1G61890	MATE efflux family protein
36	4.9	AT1G69370	CM3	chorismate mutase 3
37	4.8	AT2G38750	ANNAT4	annexin 4
38	4.8	AT1G54020	AT1G54020	GDSL-like Lipase/Acylhydrolase superfamily protein
39	4.8	AT5G47240	NUDT8	nudix hydrolase homolog 8
40	4.8	AT3G15790	MBD11	methyl-CPG-binding domain 11
41	4.7	AT3G50280	AT3G50280	HXXXD-type acyl-transferase family protein
42	4.6	AT4G01080	TBL26	TRICHOME BIREFRINGENCE-LIKE 26
43	4.6	AT2G34810	AT2G34810	FAD-binding Berberine family protein
44	4.6	AT4G24380	AT4G24380	dihydrofolate reductase
45	4.5	AT3G25780	AOC3	allene oxide cyclase 3
46	4.5	AT5G53050	AT5G53050	alpha/beta-Hydrolases superfamily protein
47	4.5	AT5G55120	VTC5	GDP-L-galactose phosphorylase VITAMIN C DEFECTIVE 5 (VTC5)
48	4.5	AT4G36950	MAPKKK21	mitogen-activated protein kinase kinase kinase 21
49	4.5	AT1G74930	ORA47	Integrase-type DNA-binding superfamily protein
50	4.4	AT2G20340	AAS	Pyridoxal phosphate (PLP)-dependent transferases superfamily protein

*, top 50 coexpressed genes.

Supplementary Table 5. List of the genes that are JA-inducible in wild-type but reduced JA-response in *myb47,95* double mutant leaves.

Locus	Gene	WT-JA / WT-mock		myb47,95-JA / WT-JA		myc2,3,4-JA / WT-JA		Function
		log2 FC*	FDR**	log2 FC*	FDR**	log2 FC*	FDR**	
AT1G18300	NUDT4	3.47	0.018	-3.41	0.021	-3.49	0.002	NUDIX hydrolase homolog 4
AT1G18740	B1L	1.93	0.016	-2.14	0.009	-2.41	<0.001	BYPASS 1-like, DUF793 domain containing protein
AT1G20310		3.12	<0.001	-1.90	0.024	-1.26	0.067	syringolide-induced protein
AT1G23710		2.05	0.050	-2.34	0.036	-0.93	0.350	hypothetical protein (DUF1645)
AT1G30370	DLAH	2.35	0.002	-2.18	0.017	-1.74	0.012	DAD1-like acylhydrolase
AT1G32928		2.46	0.003	-2.08	0.021	-2.86	<0.001	Avr9/Cf-9 rapidly elicited protein
AT1G52000	JAL5	3.52	0.001	-3.36	0.003	-8.30	<0.001	jacalin-related lectin 5
AT1G52030	JAL6	4.05	0.042	-6.46	0.005	-10.96	<0.001	jacalin-related lectin 6, myrosinase-binding protein 2 (MBP2)
AT1G52400	BGLU18	5.92	<0.001	-5.83	<0.001	-11.75	<0.001	beta glucosidase 18
AT1G52410	TSA1	4.95	<0.001	-3.81	<0.001	-7.42	<0.001	TSK-associating protein 1
AT1G55020	LOX1	2.46	0.016	-3.82	0.001	-2.28	0.013	lipoxygenase 1
AT1G56240	PP2-B13	2.43	0.038	-2.69	0.031	-1.76	0.085	phloem protein 2-B13
AT1G56250	PP2-B14	2.14	0.024	-2.47	0.018	-1.11	0.214	phloem protein 2-B14, VIP1-binding F-box protein (VBF)
AT1G61470	CAF1E	3.74	0.001	-2.84	0.021	-4.24	<0.001	CCR4-associated factor 1E, deadenylase
AT1G61820	BGLU46	2.60	0.023	-3.61	0.005	-7.50	<0.001	beta glucosidase 46
AT1G62540	FMO GS-OX2	1.18	0.005	-1.02	0.045	-0.23	0.658	flavin-monooxygenase glucosinolate S-oxygenase 2,
AT1G66090		1.84	0.036	-1.89	0.050	-2.07	0.004	disease resistance protein (TIR-NBS class)
AT1G66160	CMPG1	2.07	0.002	-2.73	<0.001	-3.54	<0.001	Cys, Met, Pro, and Gly protein 1
AT1G66400	CML23	2.90	<0.001	-1.72	0.034	-2.91	<0.001	calmodulin like 23
AT1G68450	PDE337	2.35	0.019	-3.55	0.007	-7.20	<0.001	pigment defective 337
AT1G72260	THI2.1	9.35	<0.001	-12.57	<0.001	-12.57	<0.001	thionin 2.1, a cysteine rich protein having antimicrobial properties
AT1G74450	ROH1D	2.22	0.012	-1.91	0.047	-1.90	0.008	ROH1D
AT1G76600		2.48	0.020	-2.34	0.045	-0.28	0.825	PADRE protein up-regulated after infection by S. sclerotiorun
AT1G79075	SORF17	1.81	0.023	-1.85	0.042	-0.90	0.248	short open reading frame
AT2G15220		2.42	0.005	-3.94	<0.001	-1.68	0.038	plant basic secretory protein (BSP) family protein
AT2G22500	UCP5	1.92	0.033	-1.97	0.041	-2.24	0.002	uncoupling protein 5, dicarboxylate carrier 1 (DIC1)
AT2G22880	VQ12	2.30	0.001	-1.82	0.018	-2.92	<0.001	VQ motif containing protein 12
AT2G23270	PIP3	2.98	0.004	-3.54	0.001	-0.54	0.653	pathogen-associated molecular patterns (PAMP)-induced secreted peptide 3
AT2G25735		2.36	<0.001	-1.46	0.034	-1.74	0.001	hypothetical protein
AT2G30020	AP2C1	3.45	0.002	-2.35	0.046	-3.53	<0.001	Arabidopsis Ser/Thr phosphatase of type 2C 1
AT2G31990		0.95	0.034	-1.14	0.022	-0.36	0.454	exostosin family protein
AT2G32130	CHIQL7	4.67	<0.001	-2.36	0.045	-4.65	<0.001	CHIQUITA1-like 7
AT2G32235		3.84	0.002	-3.17	0.024	-2.70	0.015	hypothetical protein
AT2G35930	PUB23	1.94	0.039	-2.33	0.019	-2.30	0.003	plant U-box 23
AT2G36220		1.89	0.001	-1.59	0.016	-2.24	<0.001	hypothetical protein
AT2G37430	ZAT11	2.17	0.018	-2.71	0.006	-0.52	0.613	zinc finger of Arabidopsis thaliana 11
AT3G01830		3.13	0.003	-2.97	0.005	-4.11	<0.001	calcium-binding EF-hand family protein
AT3G02840		3.87	0.013	-3.55	0.021	-3.40	0.005	ARM repeat superfamily protein
AT3G10930	IDL7	3.77	0.005	-2.98	0.036	-3.43	0.002	IDA-like 7, encodes a small secreted signaling peptide
AT3G15810		0.92	0.037	-1.01	0.043	-1.10	0.003	LURP-one-like protein (DUF567)
AT3G16470	JAL35	4.09	<0.001	-2.68	0.018	-6.64	<0.001	jacalin-related lectin 35, jasmonate responsive 1 (JR1)

AT3G19580	AZF2	1.61	0.008	-1.56	0.021	-1.19	0.028	zinc finger protein 2, Arabidopsis zinc finger protein 2
AT3G23170	HUP39	2.74	0.002	-2.13	0.032	-2.64	<0.001	hypoxia response unknown protein 39
AT3G23250	MYB15	2.85	<0.001	-2.42	0.001	-2.94	<0.001	MYB domain protein 15, key regulator of lignin biosynthesis in immunity
AT3G28210	PMZ	2.42	<0.001	-1.26	0.041	-1.94	<0.001	putative zinc finger protein
AT3G28220		7.56	<0.001	-7.23	<0.001	-12.89	<0.001	TRAF homology (MATH) domain containing protein
AT3G28290	AT14A	8.89	<0.001	-4.58	0.035	-11.87	<0.001	Arabidopsis thaliana 14a
AT3G28340	GATL10	2.77	<0.001	-1.80	0.041	-2.93	<0.001	galacturonosyltransferase-like 10
AT3G32980		1.88	0.001	-2.88	<0.001	-4.02	<0.001	peroxidase superfamily protein
AT3G46080		2.73	0.009	-3.00	0.006	-2.28	0.011	C2H2-type zinc finger family protein
AT3G46620	RDUF1	2.84	0.008	-2.53	0.029	-2.83	0.002	RING and DUF1117 domain-containing protein 1
AT3G53600	ZAT18	3.70	<0.001	-2.67	0.007	-5.08	<0.001	zinc finger protein
AT3G56790		2.29	0.029	-3.21	0.004	-2.57	0.004	RNA splicing factor-like protein
AT3G61190	BAP1	3.14	0.006	-3.13	0.009	-4.15	<0.001	BON association protein 1, encodes a protein with a C2 domain
AT4G01360	BPS3	2.73	0.034	-2.76	0.034	-1.35	0.215	BYPASS 3
AT4G16500	CYS4	1.52	0.019	-1.74	0.014	-2.42	<0.001	phytocystatin 4
AT4G25810	XTH23	1.62	0.011	-2.00	0.004	-2.92	<0.001	xyloglucan endotransglucosylase/hydrolase protein 23
AT4G27652		3.14	0.012	-2.91	0.029	-3.83	<0.001	hypothetical protein
AT4G27654		4.49	0.006	-3.37	0.040	-5.09	<0.001	transmembrane protein
AT4G27852		1.42	0.002	-1.42	0.008	-2.11	<0.001	natural antisense transcript overlaps with AT4G27850 and AT4G27860
AT4G27870	MEB3	0.79	0.046	-1.19	0.003	-1.21	<0.001	membrane of ER body 3, vacuolar iron transporter (VIT) family protein
AT4G37070	PLP1	2.27	0.001	-6.03	<0.001	-5.59	<0.001	patatin-like protein 1, phospholipase A IVA (PLA-IVA)
AT4G39670	GLTP	3.06	<0.001	-2.30	0.009	-2.88	<0.001	phospholipase-like protein, glycolipid transfer protein (GLTP) superfamily
AT5G05300	IDL6	2.04	0.032	-2.11	0.045	-2.19	0.007	IDA-like 6, pathogen-associated molecular patterns (PAMP)-induced
AT5G09876		1.87	0.040	-2.21	0.034	-2.36	0.003	hypothetical protein
AT5G12880		1.61	0.001	-1.52	0.006	-1.16	0.012	proline-rich family protein
AT5G22250	CAF1B	3.02	<0.001	-1.83	0.020	-2.46	<0.001	CCR4-associated factor 1B, mRNA deadenylation activity.
AT5G22520		2.67	0.006	-2.26	0.041	-2.29	0.007	hypothetical protein
AT5G24290	MEB2	2.93	<0.001	-1.97	0.021	-4.52	<0.001	membrane of ER body 2, vacuolar iron transporter (VIT) family protein
AT5G24420	PGL5	6.43	<0.001	-5.87	<0.001	-10.14	<0.001	6-phosphogluconolactonase 5
AT5G42380	CML37	3.36	0.024	-3.91	0.006	-3.05	0.008	calmodulin like 37
AT5G44610	MAP18	3.71	0.010	-5.03	0.005	-6.88	<0.001	microtuble-associated protein 18
AT5G57510		4.09	<0.001	-2.76	0.002	-3.89	<0.001	cotton fiber protein
AT5G58680		3.72	<0.001	-1.62	0.047	-4.15	<0.001	ARM repeat superfamily protein
AT5G59820	ZAT12	3.45	<0.001	-2.17	0.017	-3.09	<0.001	responsive to high light 41 (RHL41), zinc finger protein 12
AT5G64660	CMPG2	1.60	0.037	-2.07	0.008	-2.04	0.001	Cys, Met, Pro, and Gly protein 2
AT5G65300	SUPA	3.44	0.001	-3.15	0.004	-3.84	<0.001	salt up-regulated gene A, response to a variety of biotic and abiotic stresses.

The red rows show ER body-related genes, and the blue rows show defence-related genes. *, log2 fold changes. **, False discovery rate (FDR) adjusted p-values.

Supplementary Table 6. List of the genes that are downregulated in response to JA in wild-type but show less downregulation in *myb47,95* double mutant leaves.

Locus	Gene	WT-JA / WT-mock		myb47,95-JA / WT-JA		myc2,3,4-JA / WT-JA		Function
		log2 FC*	FDR**	log2 FC*	FDR**	log2 FC*	FDR**	
AT1G52700		-1.61	0.039	1.83	0.038	-2.01	0.006	alpha/beta-hydrolases superfamily protein
AT1G76530	PILS4	-5.26	<0.001	4.10	<0.001	2.85	0.006	PIN-LIKES 4, auxin transport facilitator
AT2G26080	GLDP2	-1.63	0.005	1.45	0.033	0.91	0.105	glycine decarboxylase P-protein 2, P-subunit of glycine decarboxylase
AT3G48280	CYP71A25	-1.37	0.018	1.63	0.009	1.45	0.004	cytochrome P450
AT4G12320	CYP706A6	-1.93	0.002	1.51	0.042	2.52	<0.001	cytochrome P450
AT4G24670	TAR2	-1.33	0.045	1.58	0.032	0.40	0.594	tryptophan aminotransferase related 2
AT4G33010	GLDP1	-1.86	0.001	1.56	0.020	0.80	0.165	glycine decarboxylase P-protein 1, P-subunit of glycine decarboxylase
AT4G35090	CAT2	-1.38	0.024	1.66	0.014	0.22	0.778	catalase 2
AT5G14820		-2.13	0.003	1.97	0.020	1.63	0.017	Pentatricopeptide repeat (PPR) superfamily protein
AT5G35777		-1.96	0.045	2.58	0.016	1.95	0.025	copia-like retrotransposon family
AT5G58310	MES18	-3.28	0.001	2.60	0.027	0.35	0.792	methyl esterase 18, methyl IAA esterase activity
AT5G62170	TRM25	-2.07	0.012	2.17	0.021	2.18	0.003	TON1 reducing motif 25, M-phase inducer phosphatase-like protein

The red rows show ER body-related genes, and the blue rows show defence-related genes. *, log2 fold changes. **, False discovery rate (FDR) adjusted p-values.

Supplementary Table 7. Quantification of glucosinolate contents of leaves by liquid chromatography-mass spectrometry (LC-MS).

Glucosinoalte type	Wild type		*myb47 myb95*		*myc2 myc3 myc4*	
	mock	JA	mock	JA	mock	JA
**Aliphatic**						
n-hexyl	35024±4568 [a]	34268±6529 [a]	28978±5726 [a]	41394±11806 [a]	32±7 [b]	18±7 [b]
n-heptyl	60033±7436 [a]	64978±12228 [a]	52513±6759 [a]	73065±21431 [a]	50±11 [b]	55±11 [b]
3-methylthiopropyl	9504±592 [b]	64995±10153 [a]	12093±2711 [b]	79711±14446 [a]	77±9 [b]	59±11 [b]
4-methylthiobutyl	14770±1511 [a]	22114±2764 [a]	14993±2537 [a]	23173±5087 [a]	24±6 [b]	21±7 [b]
5-methylthiopentyl	40924±4400 [a]	52085±8025 [a]	41126±7202 [a]	49585±13787 [a]	27±9 [b]	27±6 [b]
6-methylthiohexyl	70575±8413 [a]	90819±14175 [a]	78040±6452 [a]	89645±20880 [a]	29±8 [b]	38±9 [b]
7-methylthioheptyl	339372±38293 [a]	352421±54814 [a]	368093±29028 [a]	357302±78827 [a]	37±9 [b]	25±6 [b]
8-methylthiooctyl	660358±64402 [a]	679005±81726 [a]	685877±42753 [a]	619417±87876 [a]	69±18 [b]	48±10 [b]
3-methylsulfinylpropyl	7705±730 [ab]	5651±345 [ab]	9069±1164 [a]	6165±1037 [ab]	1503±192 [c]	913±376 [c]
4-methylsulfinylbutyl	57739±5272 [a]	31617±2877 [b]	68576±4403 [a]	35243±5370 [b]	66±11 [c]	64±10 [c]
8-methylsulfinyloctyl	285568±34678 [a]	152497±26484 [b]	319573±37648 [a]	129965±31426 [b]	98±22 [c]	99±37 [c]
8-methylsulfonyloctyl	2992±424 [a]	2989±412 [a]	2220±279 [a]	2866±326 [a]	126±23 [b]	86±27 [b]
**Indolic**						
3-indolylmethyl	278386±38746 [b]	523300±82047 [a]	325805±35131 [b]	334779±54695 [ab]	1860±333 [c]	3840±2265 [c]
1-methoxy-3-indolylmethyl	33994±3275 [a]	11719±1999 [bc]	38823±2181 [a]	16322±3924 [b]	2730±218 [c]	3538±1442 [c]
4-methoxy-3-indolylmethyl	252230±29894 [a]	116735±19990 [bc]	269852±11588 [a]	142541±29817 [b]	27130±813 [d]	29489±13573 [cd]
4-hydroxy-3-indolylmethyl	9234±710 [bc]	27340±4394 [a]	11729±1701 [b]	13883±2153 [b]	604±85 [c]	631±302 [c]

The values show an average ± standard error (SE) of six biological replications. Different lowercase letters indicate significant differences ($p < 0.05$; Tukey's test).

Supplementary Table 8. Nucleotide sequences of oligonucleotide primers used in this study.

Name	Sequence (5' to 3')	note
TSA1-qPCR-LP	AAGTTGCCACGGAGAAGCAAAC	qRT-PCR
TSA1-qPCR-RP	CGCCAACAGTTTCTTGATTGCG	
BGLU18-qPCR-LP	AAAGCCCAAGGACCTGTTTGC	qRT-PCR
BGLU18-qPCR-RP	AAATGCTGCTGTTGCGGTTG	
VSP2-qPCR-Fw	CGTCGATTCGAAAACCATCT	qRT-PCR
VSP2-qPCR-Rv	GGCACCGTGTCGAAGTCTAT	
MYB47-qPCR-F	TGGTTGGCTGGAGAAAATGG	qRT-PCR
MYB47-qPCR-R	AGGGAACGCCAATCACAAAC	
MYB95-qPCR-F	AAACAGGTGGGCAGCAATAG	qRT-PCR
MYB95-qPCR-R	GGCTTGTTGTTGTTGTTGGC	
MYC2-qPCR-F	GCGATGAAGGTAAACGAAGCTC	qRT-PCR
MYC2-qPCR-R	AGTGGCTCTTCTCTACCGTTTG	
MYC3-qPCR-F	TGTATCGCGACTCAAAACGG	qRT-PCR
MYC3-qPCR-R	TGTTTCCACCACCGTTGTTG	
MYC4-qPCR-F	TGTTGTTAGGTTGGGGAGATGG	qRT-PCR
MYC4-qPCR-R	ATGCTCTTGTTCAGCTGCAC	
UBQ10-Q2-F	GAAGTGGAAAGCTCCGACAC	qRT-PCR/RT-PCR
UBQ10-Q2-R	TTAGAAACCACCACGAAGACG	
MYB47 CDS-F	ATGGGGAGGACGACATGG	cDNA cloning/RT-PCR
MYB47 CDS-R	TCAAAAGAGATGATCAAGTATGT	
MYB95 TOPO-F	CACCATGGGGAGGACGACGTGG	cDNA cloning/RT-PCR
MYB95 TOPO-R	TCAAAGAAGGAACAGGTCAAGG	
MYC2 TOPO-F	CACCATGACTGATTACCGGCTAC	cDNA cloning
MYC2 TOPO-R	TTAACCGATTTTTGAAATCAAAC	
MYC3 TOPO-F	CACCATGAACGGCACAAACATCAT	cDNA cloning
MYC3 TOPO-R	TCAATAGTTTTCTCCGACTTTCG	
MYC4 TOPO-F	CACCATGTCTCCGACGAATGTTC	cDNA cloning
MYC4 TOPO-R	TCATGGACATTCTCCAACTTTCT	
pTSA1HindIIIF	AGACAAGCTTGTCTGTCCATGGATTGATAT	promoter cloning
pTSA1BamHIR	ACAGGGATCCAGCTTGAAGAAGCATCAC	
Biotin-pTSA1-F	Biotin-GAATGAAATCCACGTTTAAGTTAGTTACAACGGTTT	
pTSA1-F	GAATGAAATCCACGTTTAAGTTAGTTACAACGGTTT	EMSA
pTSA1-R	AAACCGTTGTAACTAACTTAAACGTGGATTTCATTC	
mbox1-pTSA1-F	GAATGAAATCTTTCCCCAAGTTAGTTACAACGGTTT	EMSA & promoter mutagenesis
mbox1-pTSA1-R	AAACCGTTGTAACTAACTTGGGGAAAGATTTCATTC	
mbox2-pTSA1-F	GAATGAAATCCACGTTTAACCCCTTTACAACGGTTT	EMSA & promoter mutagenesis
mbox2-pTSA1-R	AAACCGTTGTAAAGGGGTTAAACGTGGATTTCATTC	
mbox1box2-pTSA1-F	GAATGAAATCTTTCCCCAACCCCTTTACAACGGTTT	EMSA & promoter mutagenesis
mbox1box2-pTSA1-R	AAACCGTTGTAAAGGGGTTGGGGAAAGATTTCATTC	
myb47-2-LP	CCTCTTTTGCTTGATCACTGC	gentyping
myb47-2-RP	TTTTGCAACCAAATCAAGAATG	
myb47-2-Lb	TGATGGTTCACGTAGTGGGCCATCG	gentyping (T-DNA primer LBa1)
myb95-1-LP	TATGAAAGCCAAGTGTTTCCC	gentyping
myb95-1-RP	GGAACAGGTCAAGGTGTTCAG	
myb95-2-Lb	ATATTGACCATCATACTCATTGC	gentyping (T-DNA primer o8409)
myb47-2-qPCR-F	GAGCTGCAGATTAAGGTGGC	qRT-PCR for *myb47-2*
myb47-2-qPCR-R	CGTGGGTCATAGGGTCGATT	
myb95-1-qPCR-F	AGGCAAGTTCACTCCTCAGG	qRT-PCR for *myb95-1*
myb95-1-qPCR-R	GGCTTGTTGTTGTTGTTGGC	

Supplementary Table 9 Accession numbers of proteins used in the phylogenetic analysis.

ID	Gene name/annotation	Plant species	Family	Order	Subclade in Suppl. Fig. 16	Subclade in Suppl. Fig. 17
TAIR (http://www.arabidopsis.org)						
AT3G27920.1	MYB0/GLABROUS1	*Arabidopsis thaliana*	Brassicaceae	Brassicales	(outgroup)	(outgroup)
AT1G06180.1	MYB13	*Arabidopsis thaliana*	Brassicaceae	Brassicales	(outgroup)	-
AT5G15310.1	MYB16	*Arabidopsis thaliana*	Brassicaceae	Brassicales	Angiosperm MYB16/106/MIXTA	(outgroup)
AT3G01140.1	MYB106	*Arabidopsis thaliana*	Brassicaceae	Brassicales	Angiosperm MYB16/106/MIXTA	-
AT5G61420.2	MYB28	*Arabidopsis thaliana*	Brassicaceae	Brassicales	Brassicales Glucosinolate MYBs	MYB28/29/76
AT5G07690.1	MYB29	*Arabidopsis thaliana*	Brassicaceae	Brassicales	Brassicales Glucosinolate MYBs	MYB28/29/76
AT5G07700.1	MYB76	*Arabidopsis thaliana*	Brassicaceae	Brassicales	Brassicales Glucosinolate MYBs	MYB28/29/76
AT5G60890.1	MYB34	*Arabidopsis thaliana*	Brassicaceae	Brassicales	Brassicales Glucosinolate MYBs	MYB34/51/122
AT1G18570.1	MYB51	*Arabidopsis thaliana*	Brassicaceae	Brassicales	Brassicales Glucosinolate MYBs	MYB34/51/122
AT1G74080.1	MYB122	*Arabidopsis thaliana*	Brassicaceae	Brassicales	Brassicales Glucosinolate MYBs	MYB34/51/122
AT1G18710.1	MYB47	*Arabidopsis thaliana*	Brassicaceae	Brassicales	Brassicales MYB47/95	MYB47/95
AT1G74430.1	MYB95	*Arabidopsis thaliana*	Brassicaceae	Brassicales	Brassicales MYB47/95	MYB47/95
GenBank (https://www.ncbi.nlm.nih.gov/genbank/)						
MCE2055643.1		*Datura stramonium*	Solanaceae	Solanales	Angiosperm MYB16/106/MIXTA	-
KAL3336922.1	AABB24_029547	*Solanum stoloniferum*	Solanaceae	Solanales	Angiosperm MYB16/106/MIXTA	-
MBA0840795.1		*Gossypium armourianum*	Malvaceae	Malvales	Angiosperm MYB16/106/MIXTA	-
XP_016677671.1	MYB16	*Gossypium hirsutum*	Malvaceae	Malvales	Angiosperm MYB16/106/MIXTA	-
GLT43551.1	SLA2020_174910	*Shorea laevis*	Dipterocarpaceae	Malvales	Malvales MYB16/106/MIXTA	-
XP_017972560.1	MYB76	*Theobroma cacao*	Malvaceae	Malvales	Malvales MYB16/106/MIXTA	-
XP_010528356.1	MYB32-like	*Tarenaya hassleriana*	Cleomaceae	Brassicales	Angiosperm MYB16/106/MIXTA	-
XP_010538053.1	MYB6-like	*Tarenaya hassleriana*	Cleomaceae	Brassicales	Brassicales MYB47/95	MYB47/95
XP_010539976.1	MYB34-like	*Tarenaya hassleriana*	Cleomaceae	Brassicales	Brassicales MYB47/95	MYB47/95
XP_010547741.1	MYB34-like	*Tarenaya hassleriana*	Cleomaceae	Brassicales	Brassicales Glucosinolate MYBs	MYB34/51/122
XP_021909316.1	MYB34-like	*Carica papaya*	Caricaceae	Brassicales	Brassicales Glucosinolate MYBs	MYB34/51/122
XP_044467889.1	MYB106	*Mangifera indica*	Anacardiaceae	Sapindales	Angiosperm MYB16/106/MIXTA	-
XP_037497311.1	MYB28	*Jatropha curcas*	Euphorbiaceae	Malpighiales	Malpighiales MYBs	-
XP_021617198.1	MYB28	*Manihot esculenta*	Euphorbiaceae	Malpighiales	Malpighiales MYBs	-
XP_021642415.2	MYB29-like	*Hevea brasiliensis*	Euphorbiaceae	Malpighiales	Malpighiales MYBs	-
CAI0560488.1		*Linum tenue*	Linaceae	Malpighiales	Malpighiales MYBs	-
XP_022134455.1	MYB16-like	*Momordica charantia*	Cucurbitaceae	Cucurbitales	Angiosperm MYB16/106/MIXTA	-
CAB4274995.1		*Prunus armeniaca*	Rosaceae	Rosales	Angiosperm MYB16/106/MIXTA	-
XP_030937576.1	MYB34-like	*Quercus lobata*	Fagaceae	Fagales	Fagales MYB16/106/MIXTA	-
XP_050258037.1	MYB34-like	*Quercus robur*	Fagaceae	Fagales	Fagales MYB16/106/MIXTA	-
KAK9996111.1	SO802_020797	*Lithocarpus litseifolius*	Fagaceae	Fagales	Fagales MYB16/106/MIXTA	-
XP_003618530.1	MYB16	*Medicago truncatula*	Fabaceae	Fabales	Angiosperm MYB16/106/MIXTA	-
XP_045811375.1	MYB16-like	*Trifolium pratense*	Fabaceae	Fabales	Angiosperm MYB16/106/MIXTA	-

XP_026379337.1	MYB106-like	*Papaver somniferum*	Papaveraceae	Ranunculales	Angiosperm MYB16/106/MIXTA	-
XP_006827320.1	MYB80	*Amborella trichopoda*	Amborellaceae	Amborellales	Angiosperm MYB16/106/MIXTA	-
ASR18101.1	R2R3MYB16	*Ginkgo biloba*	Ginkgoaceae	Ginkgoales	Gymnosperm MYB16/106/MIXTA	-
QFG01313.1	MYB2	*Larix gmelinii*	Pinaceae	Pinales	Gymnosperm MYB16/106/MIXTA	-
QZZ92765.1	MYB161	*Larix kaempferi*	Pinaceae	Pinales	Gymnosperm MYB16/106/MIXTA	-
XP_057864559.1	MYB-like 4	*Cryptomeria japonica*	Cupressaceae	Cupressales	Gymnosperm MYB16/106/MIXTA	-
XP_057836365.1	MYB92	*Cryptomeria japonica*	Cupressaceae	Cupressales	Gymnosperm MYB16/106/MIXTA	-
QHG11484.1	R2R3-MYB 56	*Taxus chinensis*	Taxaceae	Cupressales	Gymnosperm MYB16/106/MIXTA	-
KAH9294679.1	KI387_038267	*Taxus chinensis*	Taxaceae	Cupressales	Gymnosperm MYB16/106/MIXTA	-
KAH9315541.1	KI387_024168	*Taxus chinensis*	Taxaceae	Cupressales	Gymnosperm MYB16/106/MIXTA	-
Putative Orthologous Groups DB (http://pogs.uoregon.edu)						
POPTR_0008s08870.1	MYB106	*Populus trichocarpa*	Salicaceae	Malpighiales	Angiosperm MYB16/106/MIXTA	-
POPTR_0008s08920.1	MYB106	*Populus trichocarpa*	Salicaceae	Malpighiales	Angiosperm MYB16/106/MIXTA	-
POPTR_0010s17300.1	MYB106	*Populus trichocarpa*	Salicaceae	Malpighiales	Angiosperm MYB16/106/MIXTA	-
POPTR_0017s12230.1	MYB16	*Populus trichocarpa*	Salicaceae	Malpighiales	Angiosperm MYB16/106/MIXTA	-
GRMZM2G032655_P01		*Zea mays*	Poaceae	Poales	Angiosperm MYB16/106/MIXTA	-
GRMZM2G111117_P01		*Zea mays*	Poaceae	Poales	Angiosperm MYB16/106/MIXTA	-
Os02g36890.1		*Oryza sativa*	Poaceae	Poales	Angiosperm MYB16/106/MIXTA	-
Os04g38740.1		*Oryza sativa*	Poaceae	Poales	Angiosperm MYB16/106/MIXTA	-
Os08g33660.1		*Oryza sativa*	Poaceae	Poales	Angiosperm MYB16/106/MIXTA	-
The 1000 Plant (1KP) transcriptome database (https://db.cngb.org/onekp/)						
TZWR-2009133		*Arabis alpina*	Brassicaceae	Brassicales	-	MYB47/95
TZWR-2010438		*Arabis alpina*	Brassicaceae	Brassicales	-	MYB34/51/122
TZWR-2006375		*Arabis alpina*	Brassicaceae	Brassicales	-	MYB28/29/76
TZWR-2004328		*Arabis alpina*	Brassicaceae	Brassicales	-	MYB28/29/76
HABV-2058102		*Draba aizoides*	Brassicaceae	Brassicales	-	MYB47/95
LAPO-2022735		*Draba oligosperma*	Brassicaceae	Brassicales	-	MYB47/95
LAPO-2026285		*Draba oligosperma*	Brassicaceae	Brassicales	-	MYB34/51/122
LJQF-2006682		*Draba ossetica*	Brassicaceae	Brassicales	-	MYB28/29/76
LJQF-2006683		*Draba ossetica*	Brassicaceae	Brassicales	-	MYB28/29/76
BXBF-2097991		*Draba sachalinensis*	Brassicaceae	Brassicales	-	MYB28/29/76
CSUV-2010524		*Cochlearea officinalis*	Brassicaceae	Brassicales	-	MYB34/51/122
CSUV-2001068		*Cochlearea officinalis*	Brassicaceae	Brassicales	-	MYB28/29/76
CSUV-2005693		*Cochlearea officinalis*	Brassicaceae	Brassicales	-	MYB28/29/76
IPWB-2009574		*Brassica nigra*	Brassicaceae	Brassicales	-	MYB47/95
IPWB-2003423		*Brassica nigra*	Brassicaceae	Brassicales	-	MYB28/29/76
IPWB-2003424		*Brassica nigra*	Brassicaceae	Brassicales	-	MYB28/29/76
IPWB-2003426		*Brassica nigra*	Brassicaceae	Brassicales	-	MYB28/29/76

VMNH-2022303	*Sinapis alba*	Brassicaceae	Brassicales	-	MYB28/29/76
QSKP-2042265	*Polanisia trachysperma*	Cleomaceae	Brassicales	-	MYB34/51/122
QSKP-2008860	*Polanisia trachysperma*	Cleomaceae	Brassicales	-	MYB28/29/76
UPZX-2001890	*Cleome viscosa*	Cleomaceae	Brassicales	-	MYB47/95
UPZX-2003091	*Cleome viscosa*	Cleomaceae	Brassicales	-	MYB28/29/76
SWPE-2002769	*Reseda odorata*	Resedaceae	Brassicales	-	MYB34/51/122
SWPE-2016231	*Reseda odorata*	Resedaceae	Brassicales	-	MYB28/29/76
UAXP-2080694	*Gyrostemon ramulosus*	Gyrostemonaceae	Brassicales	-	MYB34/51/122
RTTY-2014918	*Salvadora* sp	Salvadoraceae	Brassicales	-	MYB47/95
RTTY-2015914	*Salvadora* sp	Salvadoraceae	Brassicales	-	MYB28/29/76
DZTK-2043593	*Batis maritima*	Bataceae	Brassicales	-	MYB34/51/122
CRNC-2006320	*Limnanthes douglassii*	Limnanthaceae	Brassicales	-	MYB34/51/122
HYZL-2005508	*Akania lucens*	Akaniaceae	Brassicales	-	MYB28/29/76
