# Supplementary material for: Arabidopsis MYB47 and MYB95 transcription factors regulate jasmonate-inducible ER-body formation
Source: Commun Biol. 2025 Sep 26;8:1377. doi: 10.1038/s42003-025-08863-6 (PMC12475115; doi:10.1038/s42003-025-08863-6)
Supplement: Supplementary file 3 — Description of Additional Supplementary Files [file 42003_2025_8863_MOESM3_ESM.docx]

Description of Additional Supplementary Files

**File name:** Supplementary Data 1

**Description:** The source data for figures. The file contains the numerical data that is used to create the graphs in the figures.

**File name:** Supplementary Data 2

**Description:** Processed RNA-seq data. The file contains 1) all gene expression changes and 2) the changes of genes that are upregulated in the wild-type upon methyl jasmonate treatment. The rosette leaves from 14-d-old aseptically grown wild-type (WT), *myc2,3,4*, and *myb47,95* mutants were cut and floated on distilled water (mock) or 50 µM methyl jasmonate (JA) for 2 days. The total RNA was isolated and subjected to RNA-seq analysis. The numbers show log_2_ fold changes (log2FC) between two sample comparisons and adjusted *p*-values (FDR).
