# Supplementary material for: Arabidopsis MYB47 and MYB95 transcription factors regulate jasmonate-inducible ER-body formation
Source: Commun Biol. 2025 Sep 26;8:1377. doi: 10.1038/s42003-025-08863-6 (PMC12475115; doi:10.1038/s42003-025-08863-6)
Supplement: Supplementary file 6 — Reporting Summary [file 42003_2025_8863_MOESM6_ESM.pdf]

nature portfolio

Corresponding author(s): Kenji Yamada

Last updated by author(s): May 21, 2025

Reporting Summary

Nature Portfolio wishes to improve the reproducibility of the work that we publish. This form provides structure for consistency and transparency in reporting. For further information on Nature Portfolio policies, see our Editorial Policies and the Editorial Policy Checklist.

Statistics

For all statistical analyses, confirm that the following items are present in the figure legend, table legend, main text, or Methods section.

n/a	Confirmed	
☐	☒	The exact sample size (*n*) for each experimental group/condition, given as a discrete number and unit of measurement
☐	☒	A statement on whether measurements were taken from distinct samples or whether the same sample was measured repeatedly
☐	☒	The statistical test(s) used AND whether they are one- or two-sided<br>*Only common tests should be described solely by name; describe more complex techniques in the Methods section.*
☐	☒	A description of all covariates tested
☐	☒	A description of any assumptions or corrections, such as tests of normality and adjustment for multiple comparisons
☐	☒	A full description of the statistical parameters including central tendency (e.g. means) or other basic estimates (e.g. regression coefficient) AND variation (e.g. standard deviation) or associated estimates of uncertainty (e.g. confidence intervals)
☐	☒	For null hypothesis testing, the test statistic (e.g. *F*, *t*, *r*) with confidence intervals, effect sizes, degrees of freedom and *P* value noted<br>*Give P values as exact values whenever suitable.*
☒	☐	For Bayesian analysis, information on the choice of priors and Markov chain Monte Carlo settings
☒	☐	For hierarchical and complex designs, identification of the appropriate level for tests and full reporting of outcomes
☒	☐	Estimates of effect sizes (e.g. Cohen's *d*, Pearson's *r*), indicating how they were calculated

Our web collection on statistics for biologists contains articles on many of the points above.

Software and code

Policy information about availability of computer code

Data collection	n/a
Data analysis	n/a

For manuscripts utilizing custom algorithms or software that are central to the research but not yet described in published literature, software must be made available to editors and reviewers. We strongly encourage code deposition in a community repository (e.g. GitHub). See the Nature Portfolio guidelines for submitting code & software for further information.

Data

Policy information about availability of data

All manuscripts must include a data availability statement. This statement should provide the following information, where applicable:

- Accession codes, unique identifiers, or web links for publicly available datasets
- A description of any restrictions on data availability
- For clinical datasets or third party data, please ensure that the statement adheres to our policy

The RNA-seq data were deposited in the NCBI GEO database under the accession number GSE288027. The associated chart data and all gene response in the RNA-seq were deposited as supplemental materials.

Research involving human participants, their data, or biological material

Policy information about studies with human participants or human data. See also policy information about sex, gender (identity/presentation), and sexual orientation and race, ethnicity and racism.

Reporting on sex and gender	n/a
Reporting on race, ethnicity, or other socially relevant groupings	n/a
Population characteristics	n/a
Recruitment	n/a
Ethics oversight	n/a

Note that full information on the approval of the study protocol must also be provided in the manuscript.

Field-specific reporting

Please select the one below that is the best fit for your research. If you are not sure, read the appropriate sections before making your selection.

☒ Life sciences ☐ Behavioural & social sciences ☐ Ecological, evolutionary & environmental sciences

For a reference copy of the document with all sections, see nature.com/documents/nr-reporting-summary-flat.pdf

Life sciences study design

All studies must disclose on these points even when the disclosure is negative.

Sample size	We determined sample sizes based on practical sampling difficulty, referring to other publications, or observed variations in the preliminary analyses. We performed the experiments with at least three biological replications to conduct the statistical analyses and tried to have as many sample replications as possible. We did not use methods to predetermine the sample size.
Data exclusions	No data were excluded from the analysis unless they resulted from a serious experimental error or were irrelevant/inconclusive.
Replication	We introduced statistical analysis for all countable data with 3 to 10 biological replications. We observed multiple cells and show representatives for confocal microscope images. Immunoblots were performed two to four times to validate the reproducibility.
Randomization	The samples were allocated into experimental groups based on genotype and treatment. We did not introduce randomization but selected plant materials of similar size in the allocation to remove the variations caused by the developmental difference.
Blinding	The samples were allocated into experimental groups based on genotype and treatment. To remove the variations caused by the developmental difference, we selected plant materials of similar size for the allocation. We did not introduce randomization during sampling.

Reporting for specific materials, systems and methods

We require information from authors about some types of materials, experimental systems and methods used in many studies. Here, indicate whether each material, system or method listed is relevant to your study. If you are not sure if a list item applies to your research, read the appropriate section before selecting a response.

Materials & experimental systems

n/a	Involved in the study	
☐	☒	Antibodies
☒	☐	Eukaryotic cell lines
☒	☐	Palaeontology and archaeology
☒	☐	Animals and other organisms
☒	☐	Clinical data
☒	☐	Dual use research of concern
☐	☒	Plants

Methods

n/a	Involved in the study	
☒	☐	ChIP-seq
☒	☐	Flow cytometry
☒	☐	MRI-based neuroimaging

Antibodies

Antibodies used

1. rabitt anti-NAI2ΔSP, housemade
2. rabitt anti-BGLU18, housemade
3. rabitt anti-PYK10, housemade
4. mouse anti-GFP (JL-8), Takara Bio, Z2381N
5. mouse anti-tagRFP (RF5R), Agrisera, AS15 3028
6. mouse anti-His (His.H8), Thermo-Fisher, MA1-21315
7. mouse anti-GST (8-326), Thermo-Fisher, MA4-004

Validation

1. https://doi.org/10.1105/tpc.108.059345
2. https://doi.org/10.1093/pcp/pcp007
3. No previous validation. Validation in this study
4. https://www.takarabio.com/products/antibodies-and-elisa/fluorescent-protein-antibodies/green-fluorescent-protein-antibodies
5. https://www.agrisera.com/en/artiklar/rfp.html
6. https://www.thermofisher.com/antibody/product/6x-His-Tag-Antibody-clone-HIS-H8-Monoclonal/MA1-21315
7. https://www.thermofisher.com/antibody/product/GST-Tag-Antibody-clone-8-326-Monoclonal/MA4-004

Dual use research of concern

Policy information about dual use research of concern

Hazards

Could the accidental, deliberate or reckless misuse of agents or technologies generated in the work, or the application of information presented in the manuscript, pose a threat to:

No	Yes	
☒	☐	Public health
☒	☐	National security
☒	☐	Crops and/or livestock
☒	☐	Ecosystems
☒	☐	Any other significant area

Experiments of concern

Does the work involve any of these experiments of concern:

No	Yes	
☒	☐	Demonstrate how to render a vaccine ineffective
☒	☐	Confer resistance to therapeutically useful antibiotics or antiviral agents
☒	☐	Enhance the virulence of a pathogen or render a nonpathogen virulent
☒	☐	Increase transmissibility of a pathogen
☒	☐	Alter the host range of a pathogen
☒	☐	Enable evasion of diagnostic/detection modalities
☒	☐	Enable the weaponization of a biological agent or toxin
☒	☐	Any other potentially harmful combination of experiments and agents

Plants

Seed stocks

1. Arabidopsis thaliana Col-0 (ABRC, CS60000)
2. Arabidopsis thaliana GFP-h (from Ikuko Hara-Nishimura)
3. Arabidopsis thaliana myb47-2 (SALK_123009C), (uNASC, N680221)
4. Arabidopsis thaliana myb95-1 (GK-314F05), (uNASC, N2110975)
5. Arabidopsis thaliana myb47-2 myb95-1 (DUPLO_2725, cross of SALK_123009C and GK-314F05), (uNASC, N2110975)
6. Arabidopsis thaliana myc2 myc3 myc4 (from Henning Frerigmann)
7. Arabidopsis thaliana myb28 myb29 (from Barbara Ann Halkier)
8. Arabidopsis thaliana myb34 myb51 myb122 (from Henning Frerigmann)
9. Arabidopsis thaliana bglu18 (from ABRC, CS69084)
10. Arabidopsis thaliana nai1 (from Ikuko Hara-Nishimura)
11. Nicotiana benthamiana (commonly used laboratory line, from Ikuko Hara-Nishimura)

Novel plant genotypes

To analyse the ER body phenotype, we introduced the ER-localised GFP (SP-GFP-HDEL) gene into myb47-2, myb95-1, and myb47-2 myb95-1 lines. We used the Agrobacterium-mediated floral dip transformation method. The bacterium harbors the pBI121/SP-GFP-HDEL vector, which contains a signal peptide of pumpkin 2S albumin followed by GFP and an ER-retention signal, HDEL. The transformants were selected by GFP fluorescence, and at least two independent lines were obtained for each mutant.

Authentication

The homozygosity of each line was validated based on the genome PCR (myb47-2, myb95-1, myb47-2 myb95-1) or mutant phenotype (myc2 myc3 myc4, myb28 myb29, myb34 myb51 myb122, bglu18, nai1). We did not check second-site T-DNA insertions and genome mosaicism. The mutant plants do not show unexpected growth defect phenotypes, which may explain the less impact of second-site T-DNA insertions and genome mosaicism if they occur.
